# Adaptive drug resistance in melanoma is characterised by lipidome remodelling while maintaining cell membrane biophysical properties

**DOI:** 10.20517/cdr.2026.54

**Published:** 2026-09-16

**Authors:** Aurélie H. Benfield, Reuben S. E. Young, Grégoire J. B. Philippe, Nicholas D. Condon, Nicolau Barbosa Palma Filho, Helmut Schaider, Stephen J. Blanksby, Sónia Troeira Henriques

**Affiliations:** ^1^School of Biomedical Sciences, Centre for Microbiome Research and Centre for Immunology and Infectious Control, Faculty of Health, Queensland University of Technology, Translational Research Institute, Brisbane 4102, Australia.; ^2^Australian Research Council Centre of Excellence for Innovations in Peptide and Protein Science, Queensland University of Technology, Brisbane 4102, Australia.; ^3^Central Analytical Research Facility, Queensland University of Technology, Brisbane 4000, Australia.; ^4^Molecular Horizons and School of Science, University of Wollongong, Wollongong 2500, Australia.; ^5^Department of Chemistry, Graduate School of Science, University of Tokyo, Bunkyo-ku 113-0033, Japan.; ^6^Institute for Molecular Bioscience, The University of Queensland, Brisbane 4072, Australia.; ^7^Frazer Institute, The University of Queensland, Translational Research Institute, Brisbane 4102, Australia.; ^8^Institute of Pharmaceutical Sciences/Pharmacognosy, University of Graz, Graz 8010, Austria.; ^9^School of Chemistry and Physics, Faculty of Science, Queensland University of Technology, Brisbane 4000, Australia.

**Keywords:** Proteome, metabolomics, membrane lipids, glycerophospholipids, cholesterol, leaflet asymmetry, lipid metabolism, phospholipase digestion

## Abstract

**Aim:** Cancer drug resistance is driven by dynamic, systems-level adaptations, including cellular state transitions, epigenetic reprogramming, metabolic rewiring, and proteome remodelling. Lipid metabolic reprogramming is increasingly recognised as a contributor to resistance through alterations in the membrane composition and has been hypothesised to promote resistance by increasing membrane rigidity and limiting drug permeability. Here we tested this hypothesis in melanoma using a cell state-resolved multi-omics approach.

**Methods:** We combined lipidomics, surface-resolved phospholipid profiling, live-cell biophysical measurements, and whole-cell proteomics to characterise plasma membrane composition and function across drug-naïve, drug-tolerant persister, and permanently drug-resistant states in BRAF^V600E^ metastatic melanoma cells treated with dabrafenib.

**Results:** Lipidomic analyses revealed membrane remodelling, particularly in the drug-tolerant state, including altered fatty acid chain unsaturation, and increased cholesterol (Chol) and sphingomyelin levels. All cell states retained surface exposure of negatively charged phosphatidylserine and phosphatidylinositol. Despite compositional changes typically associated with membrane rigidification, plasma membrane fluidity and tension remain conserved across all resistance states. Proteomic analysis further revealed coordinated regulation of lipid metabolic pathways, including Chol homeostasis and sphingolipid turnover, indicating that lipid-metabolic adaptation supports survival under drug pressure without substantially altering membrane biophysical properties.

**Conclusion:** These findings show that lipid metabolic rewiring supports drug tolerance while conserving core membrane features. By resolving resistance-associated changes across both molecular and biophysical layers, this study identifies shared plasma membrane features and vulnerabilities across heterogeneous melanoma cell populations, supporting the application of membrane-active therapeutic agents and of combination strategies targeting lipid-metabolic adaptations to delay or prevent resistance.

## INTRODUCTION

Metastatic melanoma is one of the deadliest cancers worldwide due to its aggressiveness and ability to evade treatments. Targeted therapy that inhibits the BRAF-MEK pathway has significantly extended survival for many patients with BRAF-mutant tumours^[1,2]^. Despite these advances, most patients eventually relapse as tumour cells acquire drug resistance through adaptive mechanisms^[1,3,4]^. Increasing evidence suggests that, before acquiring permanent resistance, a subset of tumour cells uses a non-genetic reversible mechanism and enters a slow-cycling state known as drug-tolerant persisters (DTPs)^[5-7]^. During this phase, cells are dormant or slow-proliferating and undergo epigenetic changes that facilitate adaptation and drug tolerance. On prolonged drug exposure, DTPs regain proliferative capacity, the drug-tolerant proliferative persister (DTPP) state, finally resulting in permanent drug-resistant cells (PDRCs)^[8,9]^.

Lipids are increasingly recognised to play a crucial role in cancer biology, influencing not only the integrity of cell membranes, but also cellular metabolism, intracellular signalling, and tumour progression. Alterations in lipid metabolism and membrane lipid composition have been observed across multiple cancer types and are gaining increased attention for their role in drug resistance^[10-13]^. Multiple studies across cancer types report increased levels of sphingomyelin (SM), cholesterol (Chol) and fatty acid (FA) saturation in drug-resistant cells, leading to the hypothesis that lipid remodelling promotes resistance by rigidifying the plasma membrane and reducing drug permeability^[11,14,15]^. However, it remains unclear whether lipid compositional changes observed during drug tolerance and resistance induce functional changes in plasma membrane biophysical properties.

In this study, we investigated two BRAF^V600E^ metastatic melanoma cell models treated with dabrafenib to map the transition from drug-naïve cells through DTPs to PDRCs. We combined (i) shotgun lipidomics coupled to a selective outer leaflet digestion strategy to resolve surface-exposed lipids; (ii) live-cell measurements of plasma membrane fluidity, tension and surface charge; and (iii) whole-cell proteomics to link lipid remodelling to metabolic pathways. Using this integrated, cell surface-resolved approach, we directly tested whether lipid metabolic rewiring during adaptive drug tolerance and resistance is accompanied by changes in plasma membrane biophysical properties.

This work provides a detailed investigation of lipid remodelling across drug resistance states in melanoma and addresses a broader question in cancer biology. To our knowledge, this is the first distinction between lipids in the outer leaflet and those located intracellularly in mammalian cancer cells. Our findings reveal that the lipid species of the outer leaflet closely mirror that of the combined intracellular membrane fraction. We also showed that, while the drug tolerant state is associated with changes in lipid composition, these alterations have minimal impact on the cell membrane biophysical properties and are more likely linked to broader reprogramming of lipid metabolism to survive drug pressure.

## METHODS

### Cell culture

Metastatic melanoma cells WM164 (Rockland WM164-01-0001, RRID: CVCL_7928) and HT144 (ATCC HTB-63, RRID: CVCL_0318) were grown in tissue culture flasks containing Roswell Park Memorial Institute (RPMI) medium supplemented with 5% (v/v) fetal bovine serum (FBS; heat inactivated for 30 min at 56 °C) and 1% (v/v) penicillin/streptomycin, placed in an incubator set at 37 °C with 5% CO_2_. Cells were subcultured by dilution upon reaching ~80% confluence, every 2-3 days using 0.25% Trypsin-ethylenediaminetetraacetic acid (EDTA) solution.

The authenticity of both cell lines was verified using short tandem repeat profiling performed at the QUT Genomic Research Centre before the experiments were undertaken. Cells were regularly tested for the presence of mycoplasma using Lonza’s MycoAlert® Mycoplasma Detection kit via the mycoplasma core facility at Translational Research Institute (TRI).

### Resistance to dabrafenib

DTPs and PDRCs were generated from parental, drug-naïve melanoma cells as described before^[16,17]^. Briefly, cells were treated with 100 nM dabrafenib (GSK2118436; Selleckchem) added to the RPMI medium of drug-naïve cells at 80%-90% confluency and the medium was replaced every 3-4 days with fresh dabrafenib. Cells treated with dabrafenib for 7-30 days were classified as DTPs, while DTPPs emerged after ~30 days as distinct colonies. PDRCs were established after ≥ 105 days of continuous treatment. Permanent drug resistance was confirmed by continued proliferation following a 2-week drug holiday and dabrafenib rechallenge^[16]^.

### Extraction of structural lipids from cells

Structural lipids were extracted from drug-naïve cells, DTPs and PDRCs as before^[18]^ and previously detailed in our study^[16]^. Extractions were performed in nine samples per condition: three cell pellets (technical replicates) from each of the three independent culture flasks (biological replicates).

### Digestion of outer leaflet with phospholipase A_2_

The lipids in the outer leaflet of cell membranes were digested using a method adapted from Lorent *et al.* (2020)^[19]^. Briefly, cells were trypsinised, washed twice with Dulbecco’s Phosphate-Buffered Saline (DPBS), and resuspended at 1.2 × 10^6^ cells/mL. Aliquots of 250 µL (3 × 10^5^ cells) were dispensed into 2 mL glass vials in triplicate for the following treatments: (1) undigested control; (2) outer leaflet digestion with phospholipase A_2_ (PLA_2_); and (3) total digestion with PLA_2_. For the undigested control, cells were kept on ice. Outer leaflet digestions [treatment (2)] were performed by adding 2 µL of PLA_2_ (100 µM stock from *Apis mellifera*) directly to intact cells and samples were incubated at 37 °C for ≤ 30 min to prevent PLA_2_ from digesting the inner leaflet of the plasma membrane. For total digestion [treatment (3)], cells were lysed by 6-8 freeze-thaw cycles and 5 min of sonication. Subsequently, 2 µL of PLA_2_ was added to samples, which were incubated at 37 °C for the same duration as treatment (2). A 10 µL aliquot from each treatment was collected to assess membrane integrity using a lactate dehydrogenase (LDH) assay, as described below. PLA_2_ activity was inhibited to stop membrane digestion by adding 100 µL of stop buffer (1 M HCl in saturated NaCl, pH 1-2). Lipids were extracted immediately under ice-cold conditions using 110 µL methanol, 370 µL MTBE with 0.01% (v/v) BHT and 3 µL SPLASH LIPIDOMIX® Mass Spec Standard. To induce phase separation, samples were vortexed for 1 h and centrifuged at 2,000 *g* for 5 min at 4 °C; the organic phase was transferred to a fresh 2 mL glass vial and stored at -80 °C until analysis. All experiments were performed using at least nine samples per treatment: three cell pellets (technical replicates) from each of the three to four independent cell flasks (biological replicates).

### LDH assay

To confirm the integrity of the plasma membrane inner leaflet after PLA_2_ treatment, we measured the enzymatic activity of cytosolic LDH released into the supernatant. 10 µL aliquot from each sample was diluted with 80 µL DPBS in individual wells of a 96-well black microplate. The LDH enzymatic reaction was initiated by adding 30 µL of substrate buffer containing 600 µM reduced nicotinamide adenine dinucleotide (NADH) and 24 mM pyruvate, with a final concentration of 150 µM NADH and 6 mM pyruvate. The decrease in NADH concentration was monitored over time by measuring fluorescence intensity (λ_excitation_ = 340 nm, λ_emission_ = 460 nm) every 30 s for 30 min using a CLARIOstar microplate reader. Samples from undigested and partially digested cells with intact inner leaflets maintained a relatively high and stable fluorescence signal, indicating the absence of LDH in the medium and confirming that the membrane integrity was maintained. In contrast, sonicated, fully digested cell samples showed a rapid decline in fluorescence intensity, indicating conversion of NADH to NAD^+^ by cytosolic LDH released into the medium.

### Lipidome analysis

We used a triple quadrupole mass spectrometer (6500 QTRAP, SCIEX, ON, Canada) and methods adapted from Young *et al.* (2021)^[20]^ and detailed in our previous study^[16]^. SCIEX data files obtained from the 6500 QTRAP were processed using Lipidview® (Version 1.3 beta; SCIEX) software, including referencing biological lipids to the lipid class-based, deuterated internal standards and correction of abundances to account for naturally occurring lipid isotopes. Graphical visualisation of the data and statistical analysis were conducted using Microsoft Excel.

Drug-naïve, DTP and PDR cell samples treated with PLA_2_ showed an increased proportion of lysophospholipids and corresponding decrease in phospholipids compared to undigested samples [Supplementary Figure 1], confirming outer leaflet digestion.

### Chol quantification via derivatisation

A method adapted from Liebisch *et al.* (2006) was used to quantify free Chol from cell lipid extracts^[21]^ and previously detailed^[16]^. Chol quantification was performed using three biological replicates, each comprising derivatised extracts from three technical triplicates.

### Surface charge measurement using Zeta potential

Cells were harvested using 0.25% Trypsin-EDTA, washed twice with cold DPBS before being counted and resuspended at 2.5 × 10^5^ cells/mL in cold DPBS, and stored on ice until measurement. Disposable zeta cuvettes (Malvern, Folded capillary zeta cell) were rinsed with 100% ethanol, then ddH_2_O and finally DPBS. Cuvettes were filled with cell suspension and placed in the Zetasizer (Malvern, Zetasizer Nano ZS), where they were equilibrated for 500 s at 37 °C. Twelve measurements were acquired at a voltage of 40 V, with 50 sub-runs and 90 s pause between each run^[17]^. The experiments were performed using cell suspensions obtained from three independent cell passages or flasks.

### Fluorescence spectroscopy and generalised polarisation measurements with live cells

Melanoma cells were seeded (3 × 10^5^ cells/well) in a 96-well black microplate with a clear bottom the day before the assay. On the day of the assay, cells were washed twice with DPBS and serum- and phenol red-free RPMI medium containing 2.5 µM Di-4-ANEPPDHQ was added. Cells were incubated for 15 min at 37 °C to allow incorporation of the dye into the cell membrane, and fluorescence measurements were performed at 37 °C using a CLARIOstar microplate reader. Excitation was set to 480 nm, and emission spectra were recorded from 520 to 780 nm to observe spectral shifts and calculate the generalised polarisation (GP) using:

*GP* = (*I*_560_ - *I*_650_)/(*I*_560_ + *I*_650_)

where I_560_ and I_650_ are the emission intensities at 560 and 650 nm, respectively.

### GP measurements with large unilamellar vesicles

Large unilamellar vesicles (LUVs; diameter of 100 nm) were generated using synthetic lipids 1-palmitoyl-2-oleoyl-*sn*-glycero-3-phosphocholine (POPC), 1-palmitoyl-2-oleoyl-*sn*-glycero-3-phospho-l-serine (POPS), sheep wool Chol, and brain SM from porcine origin purchased from Avanti Polar Lipids or freshly extracted lipids from 2 × 10^6^ cells as described in section “Extraction of structural lipids from cells”. Synthetic lipids were solubilised in spectrophotometric grade chloroform and mixed at the appropriate molar ratios to produce defined model membranes at 500 µM. Fresh cell lipid extracts or model membrane lipid mixture were dried under a stream of nitrogen gas to form a lipid film in a round-bottom flask and subsequently kept in a desiccator overnight to remove residual solvent. LUVs were prepared by extrusion as previously described^[16]^ in HEPES buffer (10 mM HEPES containing 150 mM NaCl, pH 7.4) with 2.5 µM Di-4-ANEPPDHQ. Labelled LUVs were aliquoted in a 96-well black Optiplate and incubated at 37 °C for 15 min prior to performing fluorescence spectroscopy measurements, as described in the previous section “Fluorescence spectroscopy and generalised polarisation measurements with live cells”.

### Membrane tension measurements with FLIM using FLIPPER-TR dye

Melanoma cells (3 × 10^5^ cells/well) were seeded in an 8-well chambered cover glass (Nunc™ Lab-Tek™ II) and incubated overnight. On the day of the assay, serum-containing medium was removed and cells were gently washed twice with DPBS before adding phenol red- and serum-free medium containing 1 µM Flipper-TR®, a fluorescent dye to measure membrane tension in cells and tissues^[22]^. Images were acquired with a 40×/1.1NA Water HC Plan Apochromat objective on a Leica DMi8 SP8 confocal microscope with full incubation control running LAS-X software. FLIM images were captured using FALCON software and FAST FLIM files were output separately for further processing. Sufficient counts were acquired by exciting the sample with a tunable white-light laser at 480 nm, and the signal was detected between 575-625 nm, at a 400 Hz scan speed, over 20-line repetitions. Image analysis and quantification was performed using Fiji^[23]^, and utilised the BIOP-Utilities wrapper^[24]^ to generate regions of interest (ROIs) with Cellpose-SAM^[25]^. Mean intensity of each ROI was measured using a batch macro and statistical analysis was performed using GraphPad Prism (version 10.6.1).

### Two-photon excitation microscopy measurements of melanoma cells using 6-dodecanoyl-2-dimethylaminonaphthalene dye

Melanoma cells were seeded (3 × 10^5^ cells/well) the day before the assay in an 8-well chambered cover glass. On the day of the assay, cells were washed twice with DPBS before serum and phenol red-free RPMI medium containing 50 µM 6-dodecanoyl-2-dimethylaminonaphthalene (Laurdan) was added, and the microscope chamber was placed back in the incubator for 15 min at 37 °C before microscope measurements. Images were acquired with a 40×/1.3NA Oil immersion Plan Apochromat objective mounted to a Zeiss LSM710 confocal microscope and Spectra Physics MaiTai 2 photon laser running Zeiss Zen Black software. Samples were illuminated with 790 nm excitation light, and emission was detected at 400-560 and 474-532 nm. Additional brightfield images were acquired with a T-PMT using a 488 nm laser on a separate track. Images were processed and quantified as described above, with Cellpose used to generate ROIs, which were then measured for both detection windows. The ratio of the two detection windows was calculated, and statistical analysis was performed using GraphPad Prism (version 10.6.1).

### Whole-cell lysate and protein digestion for proteome analysis

Cells obtained from three biological replicates from individual flasks were lysed and digested for proteomics analysis as previously detailed^[16]^. Digested proteins were separated using an online U3000 RSLCnano nanoHPLC system with a 50 cm Easy-Spray C18 analytical column (Thermo Fisher, catalogue 160454 and ES803A) and analysed on a Q Exactive™ Plus Orbitrap™ Mass spectrometer (Thermo Scientific, USA). Raw data were processed using Proteome Discoverer (version 2.4.1.15) with protein identification performed against the *Homo sapiens* proteome (SwissProt Taxonomy 9606, database release v2020 10 18) and an internally curated protein-contaminant database.

### Statistical analysis

Data are shown as mean ± standard deviation (SD). Technical triplicates were averaged to generate a single value for each biological replicate. Biological replicates (typically *n* ≥ 3) consisted of independent flasks (DTP and PDR cells) or cell passages (drug-naïve cells). The biological replicate means (or mean proportions, where applicable) were used for statistical analyses using GraphPad Prism (version 10.6.1). To assess significant differences between variables, one-way analysis of variance (ANOVA) was used to compare more than two groups or 2-way ANOVA with Tukey’s, Dunnett’s, or Sidak’s multiple comparisons test to evaluate more than two groups with different treatments. When *P-*value ≤ 0.05, results were considered statistically significant. Lipid quantification is expressed as proportions (mol%) to provide evidence of the relative changes in membrane composition independent of cell size and total lipid content.

## RESULTS

Cancer progression and the development of drug resistance are linked to alterations in lipid composition and metabolism^[10,26,27]^. In this study, we characterised lipidomic changes that occur in metastatic melanoma cells while acquiring drug resistance, with particular attention to structural lipids that form the lipid bilayer of cell membranes [i.e., sterols, sphingolipids and glycerophospholipids (GPLs)]. The relative abundance of these lipids, together with the specific headgroups of GPLs, the length (i.e., number of carbons) and degree of unsaturation (i.e., the number of carbon-carbon double bonds) of their FA chains, modulate key biophysical properties of the membranes, including charge, fluidity, raft domain organisation, and bilayer thickness. Variations in lipid composition can influence membrane permeability and drug uptake, thereby contributing to drug resistance mechanisms, and can modulate the efficacy of membrane-targeting anticancer drugs^[10,11,15,28]^. To capture all states of resistance development (i.e., drug-naïve, DTPs, DTPPs and PDRCs), we used a protocol previously optimised^[7,16,17]^. BRAF^V600E^-positive metastatic melanoma cell lines (WM164 and HT144) were continuously exposed to 100 nM dabrafenib until they acquired permanent drug resistance (≥ 105 days) [Figure 1].

**Figure 1:**
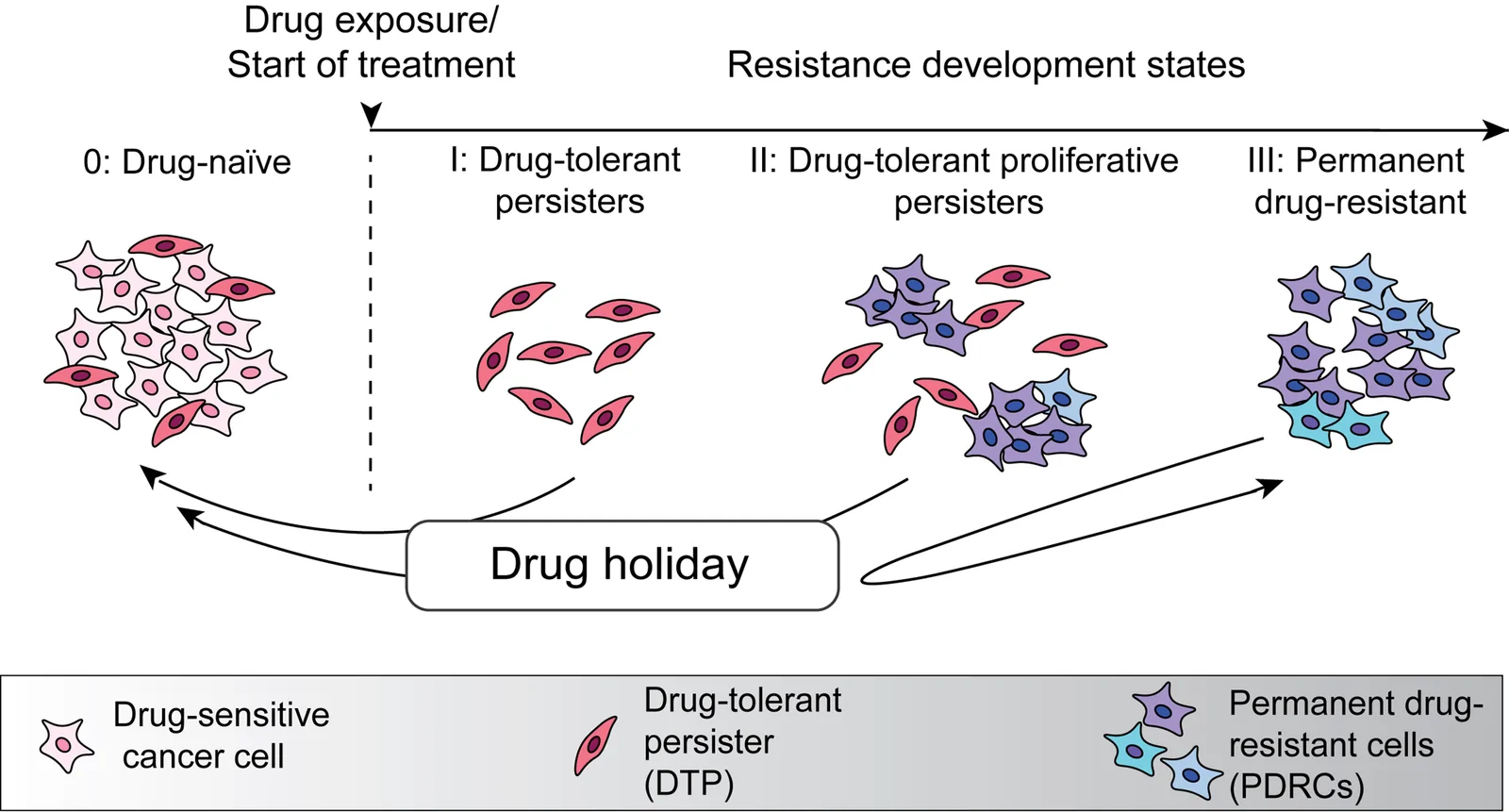
Development of permanent drug resistance to targeted therapy in (cancer) melanoma cells. Illustration depicting the progression of adaptive resistance to targeted therapy. Initially, dynamic phenotypic switching occurs between proliferative, drug-sensitive cells and slow-cycling subpopulations. DTPs can originate from pre-existing slow-cycling cells and from proliferating cells upon drug exposure. The transition into a persister state is through epigenetic reprogramming supported by metabolic adaptation and stress-response signalling. Some DTPs reinitiate proliferation (DTPPs) and permanent drug-resistant clones (PDRCs) emerge, maintaining resistance irrespective of drug withdrawal. Reproduced without modification from Benfield *et al.*^[16]^ with permission. © 2024 The Authors. DTPs: Drug-tolerant persisters; DTPPs: drug-tolerant proliferative persisters; PDRCs: permanent drug-resistant cells.

### Characterisation of lipid classes while cells acquire resistance to dabrafenib

We collected WM164 and HT144 cells at multiple time points (0, 7 days, and every 15 days between 15 and 105 days) during continuous treatment with dabrafenib, extracted their lipids and quantified the relative abundance of the four major classes of GPL [i.e., phospholipids containing phosphatidylcholine (PC), phosphatidylethanolamine (PE), phosphatidylinositol (PI), or phosphatidylserine (PS)-headgroups] and of the sphingolipid SM using a mass spectrometry-based shotgun lipidomic method as before^[17,20]^. In parallel, we quantified the Chol content using the same lipid extracts via a mass spectrometry method adapted from Liebisch *et al.* (2006)^[21]^. Untreated controls (“drug-naïve cells”) matching culture age and passage number have been previously investigated, and these major lipid classes showed no significant differences over a three-month period in culture^[16]^.

In drug-naïve WM164 and HT144 cells (Figure 2A, 0 d), PC was the predominant lipid class (57 and 59 mol%, respectively), followed by PE (21 and 15 mol%), PI (12 mol% for both cell lines), PS (7 and 9 mol%) and SM (3 and 5 mol%). After 30 days of treatment with dabrafenib, notable changes in the proportion of these lipids were observed in the persister cells (Figure 2A, 30 d). In WM164 DTPs, SM increased 3.7-fold (from 3 to 11 mol%, *P* ≤ 0.0001), negatively charged lipids (PS + PI) increased 1.2-fold (from 19 to 23 mol%), while the combined proportion of the zwitterionic GPL (PE and PC) decreased from 78 to 66 mol%^[17]^. In HT144 DTPs, SM and PE increased 1.8-fold (from 5 to 9 mol%, *P* ≤ 0.0001) and 1.3-fold (from 15 to 20 mol%, *P* ≤ 0.0001), respectively, whereas PC proportion decreased from 59 to 51 mol%. At the PDR state (105 days), the overall GPL and SM composition of PDR WM164 and HT144 cells reverted to resemble their respective drug-naïve profiles (Figure 2A, 0 d and 105 d)^[17]^.

**Figure 2:**
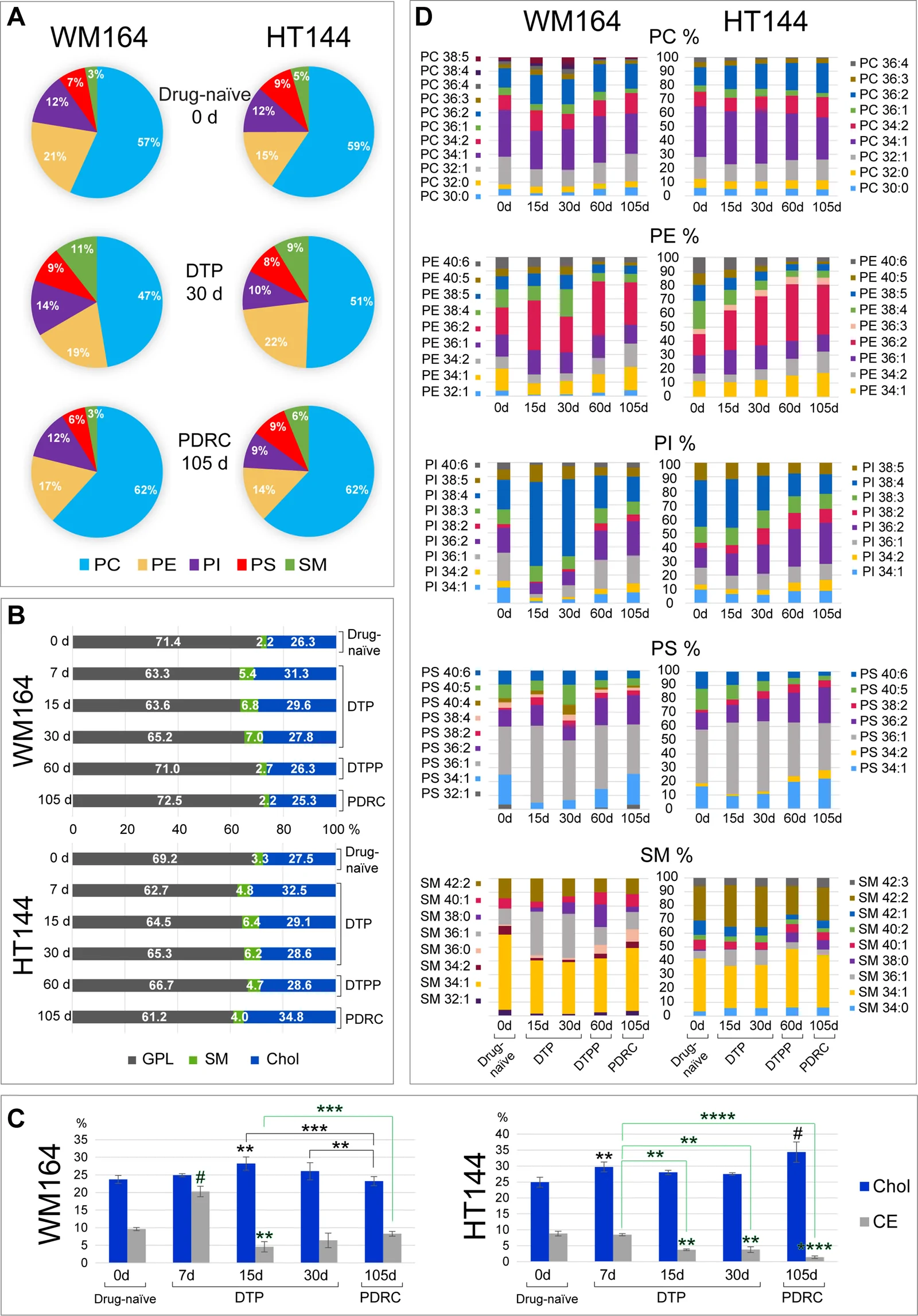
Lipid composition of WM164 and HT144 cells while acquiring resistance to dabrafenib. (A) Lipids were quantified using a shotgun-based triple quadrupole mass spectrometry approach. Pie charts show the mole percentage (mol%) of the four major GPL classes in mammalian cell membranes (i.e., PC, PE, PI and PS), and SM in WM164 and HT144 cells during treatment with dabrafenib (0, 30 and 105 days). Mean ratio displayed *n* = 3 biological replicates. See also Supplementary Figures 2 and 3; (B) Mole percentage (mol%) of GPLs (GPL = PC + PE + PS + PI), SM and Chol during incubation with dabrafenib at 0 days (drug-naïve), 7-30 days (DTP), 60 days (DTPP) and 105 days (PDRC). See also Supplementary Table 1; (C) Mole percentage (mol%) of Chol and CE relative to total lipid (GPL + SM + Chol + CE) in drug-naïve, DTP and PDRC states. Error bars represent SD. Symbols above the bars indicate values that are statistically different from the drug-naïve state (0 d): ^**^P ≤ 0.01, ^***^P ≤ 0.001, ^****^P ≤ 0.0001. “#” indicates values that are statistically different from all other states with *P* ≤ 0.0001. Symbols connected by brackets indicate statistically significant differences between the corresponding groups; (D) Histograms represent the normalised abundance of the major GPL (i.e., PC, PE, PI, PS) and SM species based on the sum composition (i.e., total number of carbons and degree of unsaturation of the two acyl chains that make up each lipid). This methodology does not distinguish lipid isomers [e.g., fatty acyl, stereospecific numbering position or double bond location(s)]. See also Supplementary File 1 for absolute values and statistical analysis using 2-way ANOVA with Dunnett’s multiple comparisons test. GPL: Glycerophospholipid; PC: phosphatidylcholine; PE: phosphatidylethanolamine; PI: phosphatidylinositol; PS: phosphatidylserine; SM: sphingomyelin; Chol: cholesterol; DTP: drug-tolerant persister; DTPP: drug-tolerant proliferative persister; PDRC: permanent drug-resistant cell; CE: cholesteryl ester; SD: standard deviation; ANOVA: analysis of variance.

The proportion of Chol and its storage form cholesteryl esters (CE) also fluctuated during acquisition of resistance [Figure 2B and C, Supplementary Table 1]. In WM164 cells, Chol content increased in DTPs, but was similar in PDRCs, whereas in HT144 cells, Chol was elevated in both DTP and PDR states compared with drug-naïve cells (see Figure 2B and Supplementary Table 1, 0, 7 and 105 d). In addition, in WM164 cells, the proportion of CE relative to total lipid increased during the first 7 days of treatment before returning to drug-naïve levels, whereas in HT144 cells, CE proportion steadily decreased as the cells acquired drug resistance [Figure 2C]. These findings suggest that development of drug resistance involves shifts in Chol and CE homeostasis, supporting the hypothesis that altered Chol metabolism plays a crucial role in regulating membrane properties and in promoting resistant cell survival.

### FA chain length and degree of saturation while acquiring resistance to dabrafenib

Because FA chain length and saturation influence membrane fluidity^[29]^, we assessed potential changes in FA chains by determining the total carbon number and degree of unsaturation (i.e., lipid sum composition) of the two FA chains in GPL and SM species during treatment of WM164 and HT144 with dabrafenib.

In WM164 cells, the acyl chain length and saturation changed within the first 30 days of treatment (Figure 2D, see 0, 15 and 30 d, Supplementary File 1). The proportion of shorter-chain FA species in all GPL classes decreased, while longer and more unsaturated species increased. For example, after 15-30 days of treatment with dabrafenib, SM 36:1 and PI 38:4 increased significantly by approximately 3-fold, whereas PI 36:1, 36:2, and 34:1 decreased (see *P*-values in Supplementary File 1). Overall, these results suggest elongation and desaturation of GPL and SM species during the DTP phase, while WM164 cells in PDR state regained a FA profile similar to drug-naïve cells^[17]^.

In HT144 cells, progressive changes in FA chain length and saturation were observed over time, becoming most evident and significantly different in PDRCs compared with drug-naïve cells [Figure 2D and Supplementary File 1]. Specifically, long polyunsaturated species (> 2 double bonds) within the PE, PI, PS classes decreased, while shorter-chain species increased proportionally. For example, PE 38:4, PI 38:4, and PS 40:5 were reduced by half, whereas PE 34:2, PE 36:2, PI 36:2 and PS 36:2 increased by 2 to 3-fold^[17]^ (see *P*-values in Supplementary File 1). Changes in PC and SM species were not statistically significant.

Overall lipidomics results show that DTPs from both cell lines have higher levels of SM, Chol, and polyunsaturated FA chains, compared with drug-naïve cells. While increases in SM and Chol are typically associated with greater membrane rigidity^[30,31]^, polyunsaturated FA chains have the opposite effect by increasing membrane fluidity^[29]^. In WM164 cells, PDRCs had a similar lipid composition to that of drug-naïve cells. In contrast, relative to drug-naïve cells, HT144 PDRCs showed reduced polyunsaturated FA chains and increased total Chol, changes typically associated with increased membrane order (rigidity).

### Lipids exposed at the surface of melanoma cells while treated with dabrafenib

To identify which GPLs are exposed on the outer leaflet of the cell membrane in metastatic melanoma cells, we adapted a method described by Lorent *et al.* (2020)^[19]^. Cells at different resistance states (i.e., drug-naïve, DTP and PDR) were incubated with PLA_2_ for 30 min to selectively digest GPLs located on the outer leaflet of the cell membrane. PLA_2_ hydrolyses the *sn*-2 ester bond of GPLs, releasing both a FA and a lysophospholipid. Intact lipids extracted after partial digestion with PLA_2_, representing those from the inner leaflet and intracellular organelles, were then compared with lipids from whole-cell extracts. The GPLs exposed on the outer leaflet were identified by subtracting the profiles of partially digested samples from those of whole-cell extracts [Figure 3].

**Figure 3:**
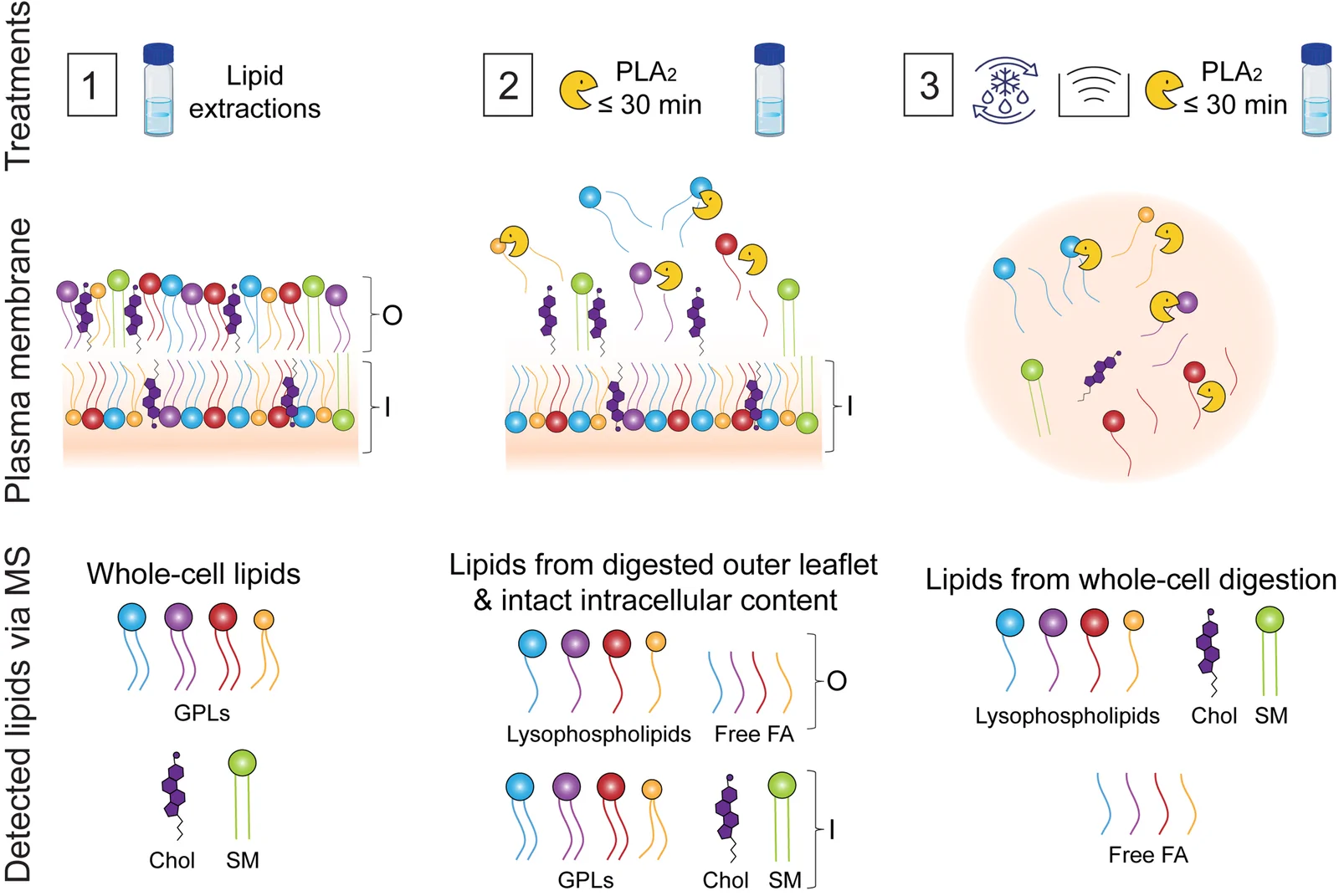
Schematic overview of treatments applied to melanoma cells prior to lipid extraction and mass spectrometry analysis to obtain outer leaflet lipid composition. Treatment 1: Phospholipids from intact/undigested cells were extracted and analysed to determine the total cellular lipidome. Treatment 2: Cells were incubated with PLA_2_ for ≤ 30 min at 37 °C to selectively digest phospholipids from the outer leaflet of the plasma membrane, while preserving the integrity of the inner leaflet, which was verified using a LDH assay (not illustrated). Extracts contain a mixture of GPLs from the plasma membrane inner leaflet and from cell organelles; lysophospholipids and free FAs from digested GPLs of the outer leaflet, and Chol and SM. Treatment 3: cells were subjected to freeze–thaw cycles, sonicated and fully digested with PLA_2_ to confirm enzymatic activity and ability to digest all the GPL classes. Treatment 3 contains lysophospholipids, FA and Chol and SM. The lipid composition of the outer leaflet (O) was obtained by subtracting the lipid profile of Treatment 2 from that of Treatment 1. This is an original figure created using Adobe Illustrator (Version 30.8). PLA_2_: Phospholipase A_2_; LDH: lactate dehydrogenase; GPLs: glycerophospholipids; FAs: fatty acids; Chol: cholesterol; SM: sphingomyelin.

Overall, all major GPL classes (i.e., PC, PE, PI and PS) were detected on the outer leaflet of both melanoma cell lines across all resistance states [Figure 4A]. Notably, the PS class, typically localised exclusively to the inner leaflet of healthy cells^[32,33]^, was also present on the outer leaflet of both melanoma cell lines, consistent with previous reports of PS externalisation in cancer cells^[34,35]^. The combined proportions of the four GPL classes were comparable between the outer leaflet and the pooled inner leaflet and intracellular compartments in DTPs and PDRCs, except for WM164 DTPs (3 mol% on the outer leaflet compared to 8 mol% on the inside of the cell). Although shifts in lipid class proportions were observed, these differences were not statistically significant [Supplementary Figure 4]. In contrast, drug-naïve cells displayed apparent distributions between the outer leaflet and the other membrane regions (Figure 4A, pie charts): WM164 cells have a higher proportion of PI and PE, and lower PS on the outer leaflet, relative to the inner leaflet and intracellular compartments, whereas HT144 cells have a higher proportion of PI and lower proportions of PE and PS.

**Figure 4:**
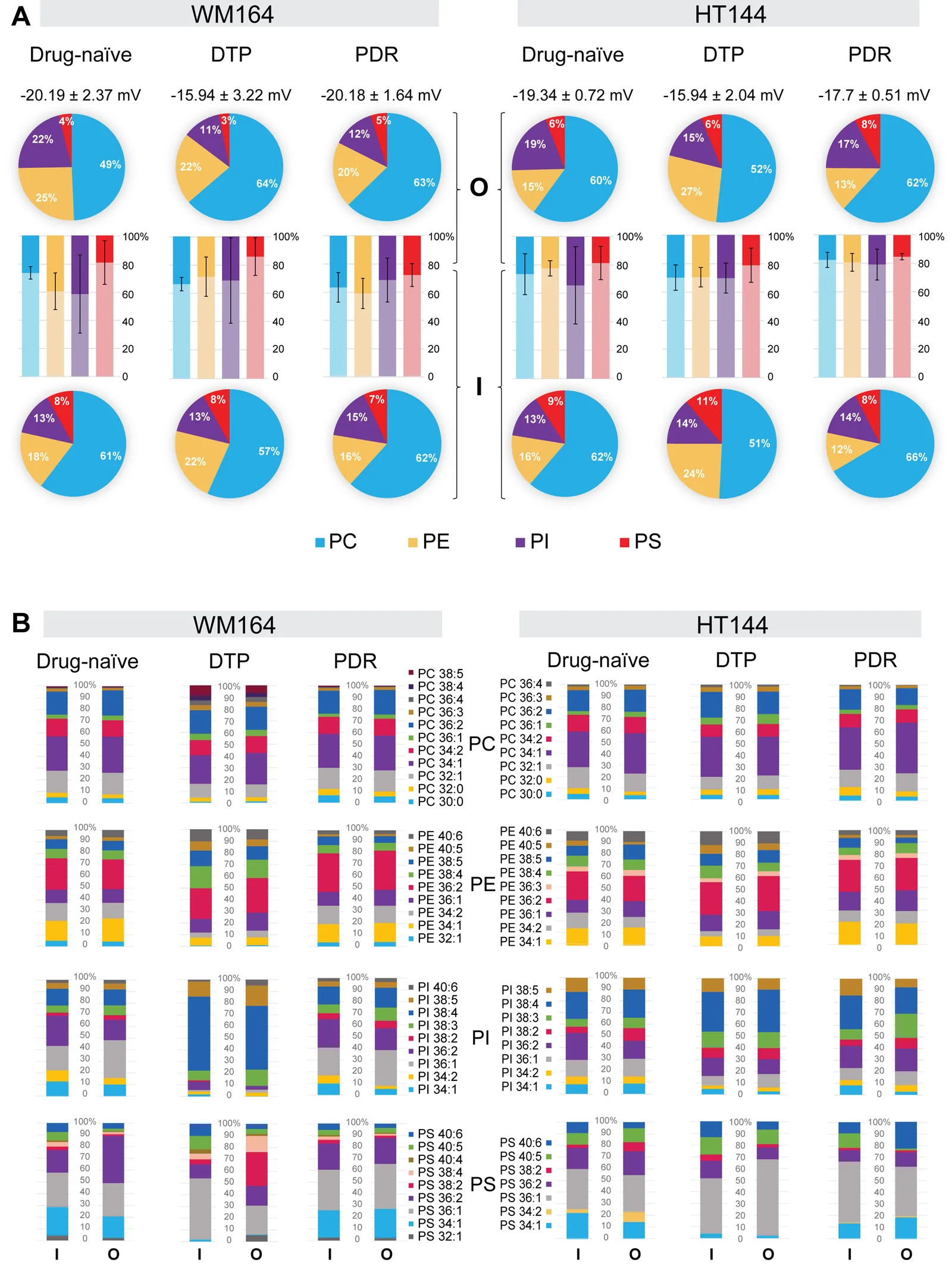
Lipids exposed at the outer leaflet of the plasma membrane of drug-naïve, DTP and PDR melanoma cells. (A) Pie charts show the mole percentage (mol%) of the four major GPL classes obtained on the outer leaflet of the plasma membrane (O, above histogram) and the lipids of the inner cytoplasmic leaflet and intracellular organelles (I, below histogram). Histograms show the proportion of each lipid class exposed on the outer leaflet (O, PC: blue, PE: yellow, PI: Purple and PS: red) and found inside the cells (I, light blue, yellow, purple and red), which include lipids of the inner leaflet combined with intracellular organelles, as quantified via mass spectrometry with error bars as SD (*n* = 3-4 biological replicates). Statistical analyses using 2-way ANOVA with Tukey’s multiple comparison test show no significant difference in the phospholipid proportions across the three different states (see also Supplementary File 2). Differences between total, outer leaflet and inner compartments within individual cell states are also not statistically significant (see also Supplementary Figure 4). Average surface charge (mV ± SD) of cells treated with dabrafenib for 0, 30 and 105 days, as measured using zeta potential (250,000 cells/sample, *n* = 3 biological replicates); differences are not statistically significant; (B) Histograms represent the normalised abundance of the most common PC, PE, PI, and PS species quantified at the sum composition level of lipids found inside cells including the inner leaflet (I) and lipids found on the outer leaflet (O). WM164 and HT144 drug-naïve, DTP and PDR states which correspond to 0, 30 and 105 days of treatment with dabrafenib. Data represent mean values from three to four separate experiments. See Supplementary File 2 for absolute values and statistical analyses using 2-way ANOVA with Sidak’s multiple comparison test. DTP: Drug-tolerant persister; PDR: permanent drug-resistant; GPL: glycerophospholipid; PC: phosphatidylcholine; PE: phosphatidylethanolamine; PI: phosphatidylinositol; PS: phosphatidylserine; SD: standard deviation; ANOVA: analysis of variance; FA: fatty acid; PDRCs: permanent drug-resistant cells.

Analysis of the FA sum composition for each GPL class in drug-naïve cells revealed differences primarily in the PS species in WM164 [Figure 4B]. In these cells, PS 36:2 comprised 39 mol% of PS species exposed on the outer leaflet, compared to 19 mol% within the inner leaflet and intracellular organelles. PS 36:1 (29 mol%) and PS 34:1 (18 mol%) were the other two major abundant outer-leaflet PS species. A modest increase in PI 36:1 was observed on the outer leaflet (32 mol%), relative to intracellular levels (21 mol%). Drug-naïve HT144 cells showed comparable proportions of lipid species detected on the outer leaflet and within the cell.

In DTP cells, changes in the FA molecular species within each lipid class were more pronounced in the outer leaflet of WM164 than in HT144 cells, which were still dividing but at a slower pace than drug-naïve cells. In WM164 DTPs, the PS and PI FA species present in the outer leaflet are distinct from those in the rest of the cell (i.e., inner leaflet and intracellular membranes). In fact, PS 38:2 was the most abundant PS species on the outer leaflet (28 mol%) but represented only 5 mol% of total cellular PS (*P* ≤ 0.0001). Conversely, PS 36:1 accounted for 24 mol% of PS on the outer leaflet yet comprised about half of the cellular PS (*P* ≤ 0.0001). PI 38:4 was the predominant PI species in the outer leaflet and on the other membranes (*P* ≤ 0.0001). Other lipid classes showed no significant variation (< 10%) between the outer and inner leaflets and intracellular compartments. In HT144 DTPs, the lipid species of the outer leaflet were similar to that of the inner leaflet and intracellular compartments, except within the PS class, where PS 36:1 made up 58% of all exposed PS compared to 37% inside the cell (*P* ≤ 0.0001). In the PDR state, the lipid species composition on the outer leaflet mirrored that of the inner leaflet and intracellular membranes, particularly for WM164. In WM164 PDRCs, the lipid profiles of the exoplasmic membrane and cell interior were similar, with comparable proportions across lipid species and only variations within the PI class. In contrast, HT144 PDRCs showed significant differences in PI (PI 38:3, 38:4, 38:5) and PS (PS 36:1, 40:5, 40:6) species, while PC and PE species were similar in the outer leaflet and the cell interior [Supplementary File 2].

Overall, the lipid profile of HT144 cells, unlike that of WM164 melanoma cells, showed no significant differences between the outer leaflet and total cellular lipid composition [Supplementary File 2]. This may reflect their distinct responses to dabrafenib: HT144 cells continued to proliferate slowly under treatment, whereas WM164 cells exhibited an even lower proliferation rate in the DTP state (7-30 days of treatment) before the emergence of highly proliferative colonies^[5]^. Nevertheless, the most pronounced differences in WM164 DTPs were the PS species in the outer leaflet, compared to inner leaflet and intracellular PS species.

### Surface charge of drug-treated melanoma cells

We investigated the cell surface charge of drug-naïve, DTP and PDR WM164 and HT144 cells by measuring their zeta potential (i.e., the electric potential at the cell interface) when treated with dabrafenib^[17]^ (Figure 4A, top of pie charts). Cancer cells are often characterised by a negative electric charge^[36]^. However, it is not known if the surface charge is maintained when cancer cells become DTPs or PDRCs.

Both drug-naïve cell types had negatively charged cell surfaces (WM164: -20.19 ± 1.37 mV, HT144: -19.34 ± 0.42 mV), which became less negative in DTPs (WM164: -15.94 ± 0.42 mV, HT144: -15.94 ± 0.59 mV) [Figure 3A]. However, a more negative surface charge was regained by PDRCs (WM164: -20.18 ± 0.95 mV, HT144: -17.7 ± 0.30 mV)^[17]^. Although the values of the surface charge were not statistically different, the zeta potential correlated with the lower amount of negatively charged lipids found on the outer leaflet of DTPs than on drug-naïve and drug-resistant cells, and the slight variations could be due to other charged molecules at the cell surface (e.g., glycosaminoglycans).

### Plasma membrane fluidity and tension in the different resistance states

Studies have shown that cancer cells can modulate their plasma membrane and become more rigid to prevent drugs from entering cancer cells^[14,37,38]^. Due to the increase in the overall amount of Chol and/or SM, we hypothesised that the WM164 DTPs and the HT144 PDRCs could be more rigid than their drug-naïve counterparts, even though the proportion of Chol and SM on the outer leaflet was not quantified. We measured the fluidity and tension of the plasma membranes of cells at the various resistance states via fluorescence spectroscopy and microscopy using several fluorescent membrane dyes such as Laurdan, Di-4-ANEPPDHQ, and FLIPPER-TR.

We used Laurdan to examine the lipid packing of the membrane of melanoma cells and measured the GP using 2-photon fluorescence microscopy [Figure 5A]. Laurdan is sensitive to the lipid membrane order by responding to the polarity of its surrounding environment within the lipid bilayer, where shifts in emission spectrum are caused by variations in membrane water content that are quantified by calculating the GP^[39]^. In general, the GP is lower in more fluid membranes, and higher in more rigid membranes. Melanoma cells labelled with Laurdan showed no significant difference in their GP measurements indicating that cell membrane lipid packing was similar between both melanoma drug-naïve, DTP and PDR cells.

**Figure 5:**
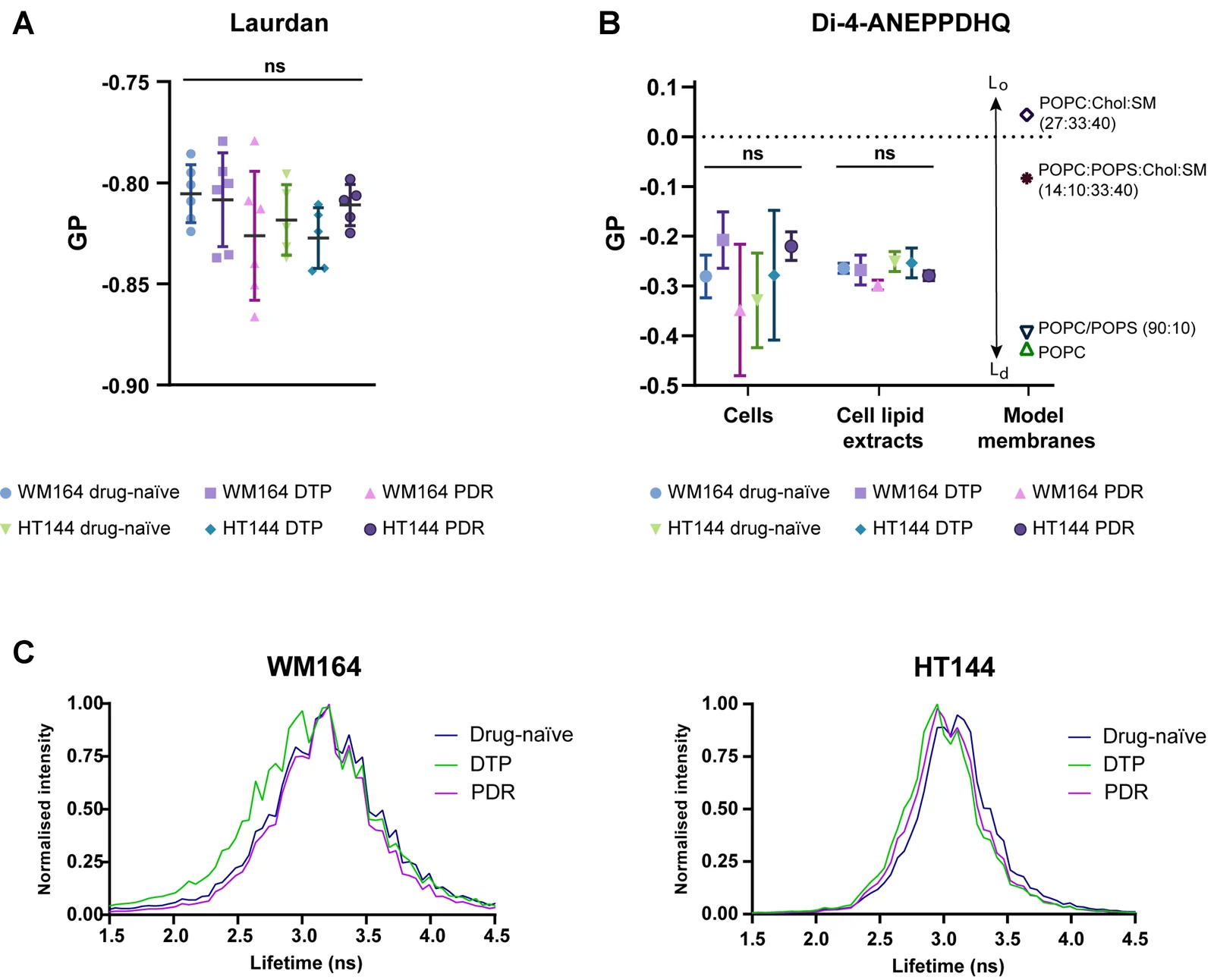
Comparing biophysical properties of melanoma drug-naïve, DTPs and PDR cell membranes using Laurdan, Di-4-ANEPPDHQ and FLIPPER-TR dye. (A) GP measurements of drug-naïve, DTP and PDR WM164 and HT144 cells labelled with Laurdan using 2-photon microscopy at 37 °C. Each point represents mean GP calculated from several cells captured in 5-7 fields of view taken per condition. The horizontal line represents the mean value and SD. One-way ANOVA with Tukey’s multiple comparison test was performed for statistical analyses. Differences are not statistically significant (ns); (B) GP measurements of WM164 and HT144 drug-naïve, DTP, and PDR melanoma cells (cells), as well as LUVs generated from melanoma cell lipid extracts or defined synthetic lipid mixtures (model membranes). Cells were plated at a density of 15,000 cells and labelled with Di-4-ANEPPDHQ for 15 min at 37 °C. LUVs prepared from cell lipid extracts or from synthetic POPC, POPC/POPS (90:10), POPC/PS/Chol/SM (17:10:33:40) and POPC/Chol/SM (27:33:40) were labelled with 2.5 µM Di-4-ANEPPDHQ at 37 °C and used as model membranes. Arrows indicate the direction of increasing membrane order, corresponding to liquid-ordered (L_o_) and liquid-disordered (L_d_) phases. Fluorescence emission spectra were measured with excitation at 480 nm. Data represent mean GP ± SD (*n* = 3-5); (C) Representative FLIM measurements of membrane tension on drug-naïve, DTP and PDR melanoma cells labelled with FLIPPER-TR probe. DTPs: Drug-tolerant persisters; PDR: permanent drug-resistant; GP: generalised polarisation; SD: standard deviation; ANOVA: analysis of variance; LUVs: large unilamellar vesicles; POPC: 1-palmitoyl-2-oleoyl-*sn*-glycero-3-phosphocholine; POPS: 1-palmitoyl-2-oleoyl-*sn*-glycero-3-phospho-l-serine; PS: phosphatidylserine; Chol: cholesterol; SM: sphingomyelin; FLIM: fluorescence lifetime imaging.

Di-4-ANEPPDHQ probe is also sensitive to cell membrane order, but its fluorescence emission is more strongly influenced by overall Chol content, charge distribution and membrane dipole potential than that of Laurdan (see model membranes in Figure 5B)^[40]^. Di-4-ANEPPDHQ was therefore used here to detect potential differences in the membrane organisation that may not be captured by Laurdan. The results show no significant difference among all three states of resistance in WM164 and HT144 cells or liposomes generated from lipids extracted from these cells, suggesting no significant changes between the membrane order of drug-naïve, DTP and PDR cells; however, a trend consistent with Chol proportions [Figure 2B] was noticeable (GP of cells in Figure 5B). These results may also reflect differences in membrane asymmetry and membrane potential rather than differences in membrane fluidity.

Although membrane fluidity and membrane tension are interdependent, they represent distinct biophysical properties. Increased membrane tension generally reduces fluidity by restricting lipid mobility and promoting a more ordered membrane state. We used the live-cell fluorescent probe FLIPPER-TR to measure membrane tension of WM164 and HT144 cells in the drug-naïve, DTP and PDR states [Figure 5C]. HT144 cells showed no significant differences in membrane tension in the three different states. In contrast, WM164 DTP cells had a slightly shorter fluorescence lifetime compared to the drug-naïve and the PDR cells, suggesting a modest decrease in membrane tension, and a slightly more fluid membrane.

The results obtained using live-cell imaging and spectroscopy with multiple membrane dyes indicate that membrane fluidity in DTPs is comparable to that of drug-naïve cells. The combined increase of Chol and SM in the DTP [Figure 2B], which would typically increase membrane rigidity, appears to be counterbalanced by the increase in polyunsaturated FA chains [Figure 2B and C, Figure 4B]. Overall, these live-cell measurements suggest that metastatic melanoma cells do not undergo major changes in membrane fluidity during development of dabrafenib resistance. However, they may still differ in other membrane features, such as transmembrane potential or microdomain organisation.

### Proteomic remodelling reveals metabolic and membrane adaptations in drug-tolerant melanoma cells

To investigate how melanoma cells remodel their proteome during resistance development, we performed whole-cell proteomic analyses on drug-naïve, DTP and PDR WM164 and HT144 cells^[17]^. Total protein extracts from dabrafenib-treated cells were compared with those from drug-naïve controls and processed following the same workflow.

Across both cell lines, DTPs exhibited a larger number of differentially expressed proteins relative to drug-naïve cells than PDRCs [Figure 6A]. Kyoto Encyclopedia of Genes and Genomes (KEGG) pathway [Figure 6B and Supplementary Figure 5]^[41-43]^ and gene ontology (GO) [Figure 6C and Supplementary Figure 6] analyses revealed enrichment of proteins implicated in drug-resistance mechanisms, including lysosomal proteins and enzymes involved in degradation of glycosaminoglycan [Figure 6B and C]. Relevant for this study, we also identified upregulation of proteins in pathways that could modulate cell membrane properties^[17]^; specifically, glycosaminoglycan degradation, glycosphingolipids and PI biosynthesis, and sphingolipid degradation.

**Figure 6:**
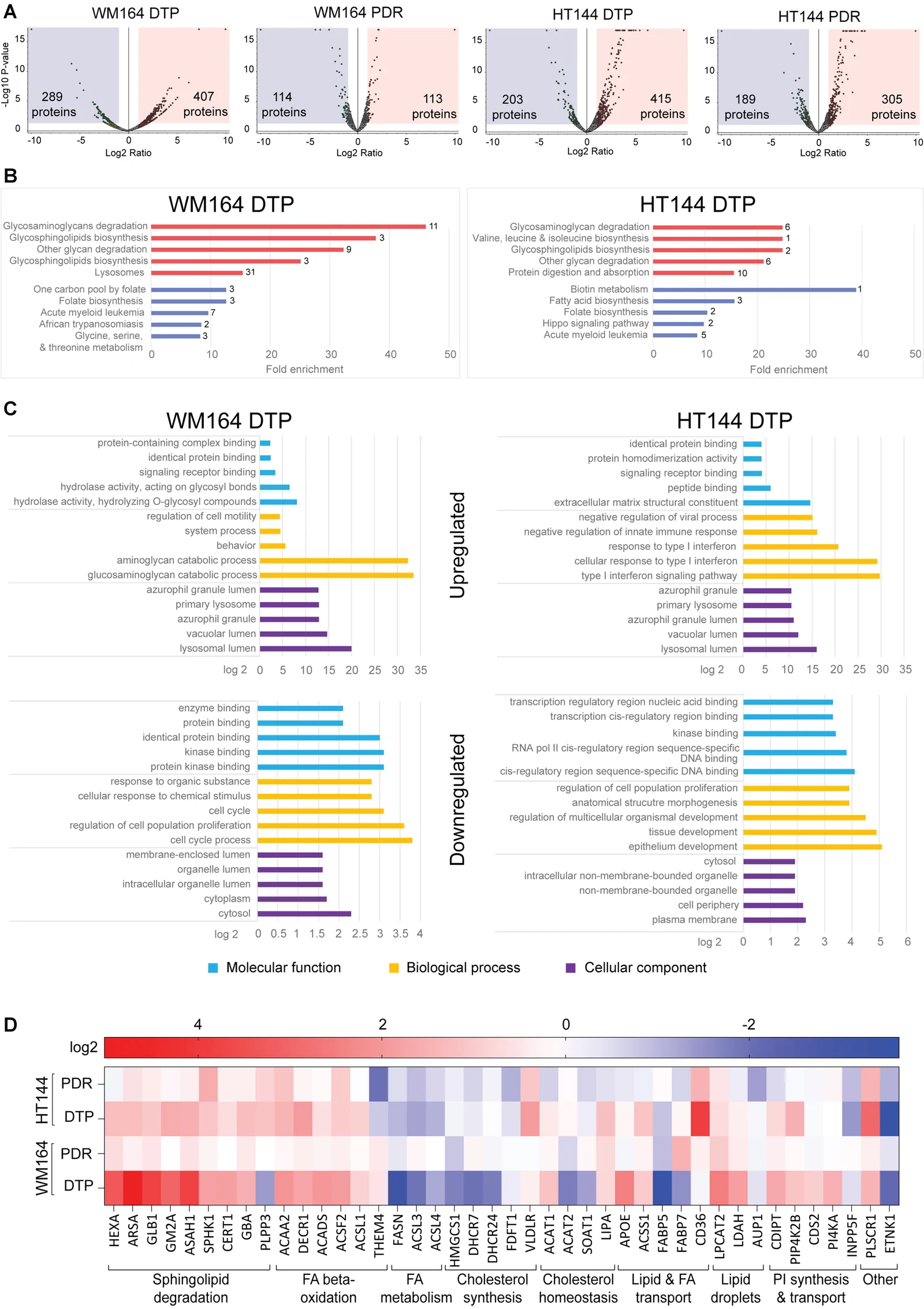
Upregulated and downregulated proteins of WM164 and HT144 cells in the persister and drug-resistant state. (A) Volcano plots of significantly downregulated (blue) and upregulated (red) proteins of WM164 and HT144 cells in a drug-tolerant state (DTP; 28 days with 100 nM dabrafenib) or a permanent drug-resistant state (PDR; ≥ 105 days with 100 nM dabrafenib) compared with untreated cells. *P*-value ≤ 0.05; log2 fold change ± 1; (B) Diagrams of WM164 and HT144 DTP with the number (end of each bar) of upregulated (red) and downregulated (blue) proteins involved in identified KEGG pathways (https://www.genome.jp/kegg/pathway.html) with *P*-value ≤ 0.05; log2 fold change ± 1 (fold enrichment: enrichment factor calculated as a quotient of number of found proteins and number of expected proteins). See also Supplementary Figure 5; (C) GO functional analysis of differentially expressed proteins in WM164 and HT144 DTPs. Proteins of DTPs were compared to proteins from drug-naïve cells. Histograms represent fold enrichment of the top 5 differentially expressed proteins (*P*-value ≤ 0.05; log2 fold change ± 1) involved in molecular function (blue), biological process (yellow) and cellular component (purple) categories. See also Supplementary Figure 6; (D) Heat map showing downregulated (blue) and upregulated (red) proteins in key lipid synthesis and metabolic pathways. Colours indicate log2 fold change of expressed proteins in WM164 and HT144 cells in the DTP or PDR states relative to drug-naïve cells. See also Supplementary File 3. DTP: Drug-tolerant persister; PDR: permanent drug-resistant; KEGG: Kyoto Encyclopedia of Genes and Genomes; GO: gene ontology; FA: fatty acid; PI: phosphatidylinositol.

Proteomics data suggest that WM164 DTPs may undergo enhanced degradation of glycosaminoglycans and other glycans, supported by the enrichment of hydrolases acting on glycosidic bonds [Figure 6B and C]^[17]^. Glycosaminoglycans play critical roles in cancer proliferation, invasion, metastasis formation, and in therapeutic resistance^[44,45]^. Given their negative charge, their degradation might also contribute to the less negative surface charge observed in the zeta potential of DTPs.

Proteins involved in glycosphingolipids biosynthesis (KEGG: hsa00603 & hsa00604) and in sphingolipids degradation (e.g., HEXA, GLB1, ARSA, ASAH1 and GBA)^[46]^ were upregulated in DTPs, but not in PDRCs [Figure 6B and C]. Sphingolipid degradation increases ceramide levels, which can be converted into SM via SM synthase^[47]^. These proteomic changes possibly contribute to the increased SM detected in the lipidome of DTPs^[17]^.

Proteins involved in FA beta-oxidation [Figure 6C and Supplementary File 3], a mitochondrial process that catabolises FA to generate energy, were also upregulated in WM164 DTPs but not in PDRCs. A similar pattern was observed in HT144 DTPs, except for the proteins ACAA2 and ACSF1 which remained upregulated in PDRCs, while THEM4 was downregulated compared to drug-naïve cells. Other proteins involved in FA metabolism, such as FASN^[48]^ and ACSL3/4, were mostly downregulated in both DTP and PDRCs, with ACSL3 being an exception^[49]^.

Several enzymes involved in PI biosynthesis, including CDIPT, CDS2, PI4KA and PIP4K2B, were upregulated in the DTP state, especially in WM164 cells [Figure 6C and Supplementary File 3]. The upregulation of CDIPT and CDS2 likely contributes to the relatively high amount of PI (PI 38:4) detected in the lipidome as both enzymes support *de novo* PI synthesis^[50,51]^. PI 38:4 also serves as the predominant precursor for PI-derived polyphosphates such as phosphatidylinositol 4,5-bisphosphate (PIP2) and phosphatidylinositol 3,4,5-trisphosphate (PIP3), which activates protein kinase B (AKT)^[52]^. In turn, AKT signalling regulates multiple pathways associated with multidrug resistance, including inhibition of apoptosis, stimulation of metabolism and cell growth^[53]^. Furthermore, the overexpression of PI4KA has been linked with cancer cell metastasis and invasion^[54]^.

Most proteins associated with Chol biosynthetic pathways (e.g., HMGCS1, DHCR7, DHCR24, FDFT1) were downregulated in WM164 DTPs, whereas apolipoprotein E (APOE), which is involved in Chol transport and lipid metabolism^[55-57]^, was strongly overexpressed^[17]^ (5.6-fold; log_2_FC = 2.48; Figure 6D and Supplementary File 3). In PDR WM164 cells, the expression level of Chol biosynthetic proteins was similar to that of drug-naïve cells (fold change 0.6-1.2). In HT144 cells, these proteins were generally downregulated in both DTPs and PDRCs (fold change 0.5-0.9), except for the very low-density lipoprotein receptor (VLDLR)^[17]^, which was upregulated [Figure 6D and Supplementary File 3]. These findings suggest that the increased Chol detected in WM164 and in HT144 DTPs is likely due to increased Chol uptake from the extracellular medium (mediated by APOE in WM164, and by VLDLR in HT144^[58]^), rather than increased *de novo* synthesis.

Other proteins involved in Chol homeostasis, including those involved in the conversion of Chol to CE (e.g., ACAT1 & 2, SOAT) and in the hydrolysis of CE to obtain Chol (e.g., LIPA), were also differentially regulated. Several aggressive cancers (e.g., melanoma, prostate, breast) upregulate Chol esterification via ACAT1 (acyl-coenzyme A: Chol acyltransferase-1)^[59]^. Consistent with this, ACAT1 was upregulated in both WM164 and HT144 DTPs (2.3 fold in WM164 and 1.4 fold in HT144). In WM164 PDRCs, ACAT1 expression was similar to that of drug-naïve cells, whereas in HT144 PDRCs it was downregulated. These patterns may explain the higher CE levels observed in WM164 DTPs and the reduced CE levels in HT144 PDRCs relative to drug-naïve cells [Figure 2C].

On the other hand, LIPA, an enzyme that breaks down CE to form Chol, was modestly upregulated (1.7 fold; see Supplementary File 3) in both WM164 DTPs and PDRCs compared to drug-naïve cells. It was similarly increased (2.3-fold) in HT144 DTPs but downregulated in HT144 PDRCs (0.7-fold). Upregulation of LIPA may contribute to elevated Chol levels detected in the WM164 DTPs. Overall, these data highlight the complexity of Chol metabolism and homeostasis in melanoma and suggest that their dynamic regulation plays a crucial role in the development of cancer resistance.

## DISCUSSION

Acquired resistance to targeted therapy in melanoma is attributed to slow-cycling DTPs that survive and adapt to sustained drug exposure^[60-62]^. Targeting DTPs is therefore critical to prevent disease progression and relapse^[63]^. We previously showed that membrane-active peptides kill drug-naïve, DTP and PDR melanoma cells with comparable efficacy, via a mechanism that involved interactions with cell membrane lipids^[16]^. Here we investigated whether lipid metabolic rewiring during the acquisition of drug resistance in melanoma is accompanied by changes in plasma membrane biophysics that could guide membrane-targeted strategies^[64,65]^.

This study demonstrates that substantial lipidomic and proteomic remodelling during adaptative drug resistance can occur without inducing major changes in plasma membrane biophysical properties. Dabrafenib treatment increased SM levels in DTPs, elevated Chol in WM164 DTPs and HT144 PDRCs, and reduced the levels of long polyunsaturated FAs in HT144 PDRCs. Such compositional changes are typically interpreted as promoting drug resistance through plasma membrane rigidification and reduced drug permeability^[10,12,66]^, as reported in lung^[67]^, breast^[11,68]^, and colorectal cancers^[69]^. However, our biophysical analyses using live cells and membrane-sensitive probes revealed minimal alterations in plasma membrane fluidity and tension across drug-naïve, DTP, and PDR melanoma cells. These findings contrast with observations in other cancer models, such as acute myeloid leukaemia, where DTPs exhibit increased membrane rigidity as a survival mechanism against chemotherapeutic agents^[70]^ as well as in chemotherapy-resistant lung cancer^[67]^ and breast cancer cells^[11]^. Together, these findings indicate that lipid remodelling during adaptative drug resistance does not necessarily induce plasma membrane rigidification, and it might vary across cancer types and resistance states.

The plasma membrane lipid class composition of the outer leaflet was distinct from the intracellular lipid class profiles of both drug-naïve cells and DTPs. Outer leaflet lipid profiling also revealed that drug-naïve, DTP and PDR cells expose PS and PI phospholipids at the cell surface. PS exposure is a well-established feature of cancer cells and has been linked to immune suppression, tumour progression, and resistance to therapy^[34,71]^. However, the externalisation of PI species to the outer leaflet remains poorly characterised and current knowledge is primarily around their phosphorylated derivatives and their role in intracellular signalling pathways, cytoskeletal reorganisation^[10,52]^ and also “eat-me” signals^[72]^ similar to PS phospholipids^[10,52,73]^.

Lipidomic and proteomic analyses support the view that adaptative drug tolerance represents a distinct cellular state phenotype, rather than an early step towards permanent resistance. DTPs display a broad metabolic difference without displaying a more rigidified cell membrane phenotype. PDR cells either reverted or had differences in the composition of the membrane, depending on the cell line, yet they also displayed conserved membrane biophysical properties. This distinction has important implications for targeting tolerant and resistant states.

Proteomic analyses provide mechanistic context for the lipidomic observations. DTPs in both melanoma models showed coordinated regulation of lipid metabolic pathways, including enzymes involved in PI biosynthesis and in sphingolipid degradation, with cell-line-specific differences in Chol homeostasis together with shifts in FA oxidation. These changes are consistent with metabolic rewiring that supports drug pressure survival implicated in melanoma^[27,74,75]^ and other cancers^[26,76-78]^, but without enforcing a rigidified membrane phenotype.

This study has some limitations. Although PLA_2_ digestion enabled surface-resolved profiling of GPL, it did not allow direct quantification of Chol or SM in the outer leaflet, limiting our ability to define the leaflet distribution of these key membrane-ordering lipids. In addition, lipid species were resolved at the sum-composition level, which does not distinguish lipid isomers, fatty acyl chain position, or location of double bonds. These structural details also influence membrane biophysical properties. While our proteomic analyses identified candidate pathways linking lipidome remodelling to adaptation during dabrafenib treatment, the mechanisms by which specific lipid alterations contribute to drug tolerance and resistance remain to be established. Future studies require novel higher-resolution, leaflet-specific approaches that can resolve Chol and SM distribution and lipid isomers. Further characterisation of membrane-domain organisation and of the functional consequences of PS and PI exposure, particularly in DTPs, will also be important. Finally, studies investigating the mechanisms of drug resistance that drive lipidome remodelling are needed to establish causal relationships between lipid alterations and therapeutic adaptation.

In summary, adaptative resistance to dabrafenib in melanoma is accompanied by lipidomic and proteomic reprogramming while preserving core cell membrane biophysical properties across drug-naïve, DTP and PDR states. This distinction between lipid composition and membrane properties reveals shared membrane vulnerabilities across heterogeneous melanoma cell populations that can be rationally exploited. Membrane-active therapeutics and delivery systems whose efficacy depends on membrane order and charge^[12,79]^ are likely to retain activity across diverse resistance states, while combination strategies targeting the lipid metabolic pathways supporting drug tolerance and resistance^[80-82]^, such as sphingolipid turnover or PI biosynthesis, offer opportunities to delay or prevent the emergence of drug resistance.

## DECLARATIONS

### Availability of data and materials

Additional information is available in the Supplementary Materials. The raw data supporting the findings of this study are available within this article and in the Supplementary Materials. Further data are available from the corresponding authors upon request.

### AI and AI-assisted tools statement

The AI tools ChatGPT (Version 5.5) and CoPilot (Version 150.0.4078.96, released 2026-07-27) were used solely for language editing during the preparation of the manuscript. The tools were not used for the study design, data collection, analysis, interpretation, or the scientific content of the work. All authors take full responsibility for the accuracy, integrity, and final content of the manuscript.

### Copyright

© The Author(s) 2026.
