# Supplementary material for: Adaptive drug resistance in melanoma is characterised by lipidome remodelling while maintaining cell membrane biophysical properties
Source: Cancer Drug Resist. 2026 Sep 16;9:35. doi: 10.20517/cdr.2026.54 (PMC13680111; doi:10.20517/cdr.2026.54)
Supplement: Supplementary file 1 [file cdr-9-35-SupplementaryMaterials.zip › cdr9054-SuplementaryMaterials/cdr9054-Supplementary Figures and Table.pdf]

Supplementary Materials

Adaptive drug resistance in melanoma is characterised by lipidome remodelling while maintaining cell membrane biophysical properties

Aurélie H. Benfield[1,2], Reuben S. E. Young[3,4], Grégoire J. B. Philippe[1,2,5], Nicholas D. Condon[6], Nicolau Barbosa Palma Filho[1,2], Helmut Schaider[7,8], Stephen J. Blanksby[3,9], Sónia Troeira Henriques[1,2,6]

[1]School of Biomedical Sciences, Centre for Microbiome Research and Centre for Immunology and Infectious Control, Faculty of Health, Queensland University of Technology, Translational Research Institute, Brisbane 4102, Australia.
[2]Australian Research Council Centre of Excellence for Innovations in Peptide and Protein Science, Queensland University of Technology, Brisbane 4102, Australia.
[3]Central Analytical Research Facility, Queensland University of Technology, Brisbane 4000, Australia.
[4]Molecular Horizons and School of Science, University of Wollongong, Wollongong 2500, Australia.
[5]Department of Chemistry, Graduate School of Science, University of Tokyo, Bunkyo-ku 113-0033, Japan.
[6]Institute for Molecular Bioscience, The University of Queensland, Brisbane 4072, Australia.
[7]Frazer Institute, The University of Queensland, Translational Research Institute, Brisbane 4102, Australia.
[8]Institute of Pharmaceutical Sciences/Pharmacognosy, University of Graz, Graz 8010, Austria.
[9]School of Chemistry and Physics, Faculty of Science, Queensland University of Technology, Brisbane 4000, Australia.

Correspondence to: Assoc. Prof. Sónia Troeira Henriques, School of Biomedical Sciences, Centre for Microbiome Research and Centre for Immunology and Infectious Control, Faculty of Health, Queensland University of Technology, Translational Research Institute, Brisbane 4102, Australia. E-mail: sonia.henriques@qut.edu.au

Supplementary Table 1. Mole ratio of glycerophospholipids (GPL), sphingomyelin (SM) and cholesterol (Chol) during incubation of WM164 and HT144 cells with 100 nM dabrafenib over 105 days and after 15 days without dabrafenib or "post-drug holiday" (PDH)

	Days with Dab	% ± SD		
		GPL	SM	Chol
WM164	0	71.4 ± 1.26	2.2 ± 0.14	26.3 ± 1.12
	7	63.3 ± 1.25***	5.4 ± 0.26	31.3 ± 1.02
	15	63.6 ± 1.79**	6.8 ± 0.36	29.6 ± 1.54
	30	65.2 ± 4.92*	7.0 ± 2.91	27.8 ± 2.04
	45	69.2 ± 3.19	4.6 ± 2.26	26.2 ± 0.93
	60	71.0 ± 3.15	2.7 ± 0.50	26.3 ± 2.98
	75	70.8 ± 3.33	2.8 ± 0.78	26.4 ± 2.68
	90	70.8 ± 3.62	2.8 ± 0.57	26.4 ± 3.06
	105	72.5 ± 1.37	2.2 ± 0.26	25.3 ± 1.21
	PDH (2 weeks)	74.1 ± 2.26	2.4 ± 0.12	23.5 ± 2.14
HT144	0	69.2 ± 1.11	3.3 ± 0.18	27.5 ± 1.20
	7	62.7 ± 1.59***	4.8 ± 0.30	32.5 ± 1.78**
	15	64.5 ± 0.64*	6.4 ± 0.10	29.1 ± 0.73
	30	65.3 ± 0.50	6.2 ± 0.52	28.6 ± 0.17
	45	66.4 ± 0.80	5.8 ± 0.27	27.8 ± 0.57
	60	66.7 ± 0.75	4.7 ± 0.43	28.6 ± 0.33
	75	61.7 ± 1.63****	5.0 ± 0.63	33.3 ± 2.22***
	90	59.3 ± 1.18****	4.9 ± 0.26	35.8 ± 0.92****
	105	61.2 ± 3.35****	4.0 ± 0.23	34.8 ± 3.35****
	PDH (2 weeks)	64.5 ± 3.77*	3.5 ± 0.30	32.0 ± 1.12*

GPL include PC, PE, PS and PI. Mean ratio ± SD displayed (n = 3 biological replicates). Statistical differences in GPL, SM and Chol ratios were assessed by two-way ANOVA (Tukey's multiple comparison) with drug-naïve cells (0 days with dabrafenib) as a reference unless otherwise indicated with bracket and indicated as **P*≤0.05, ***P*≤0.01, ****P*≤0.001, *****P*≤0.0001. All other differences were not significant. Related to Figure 2B.

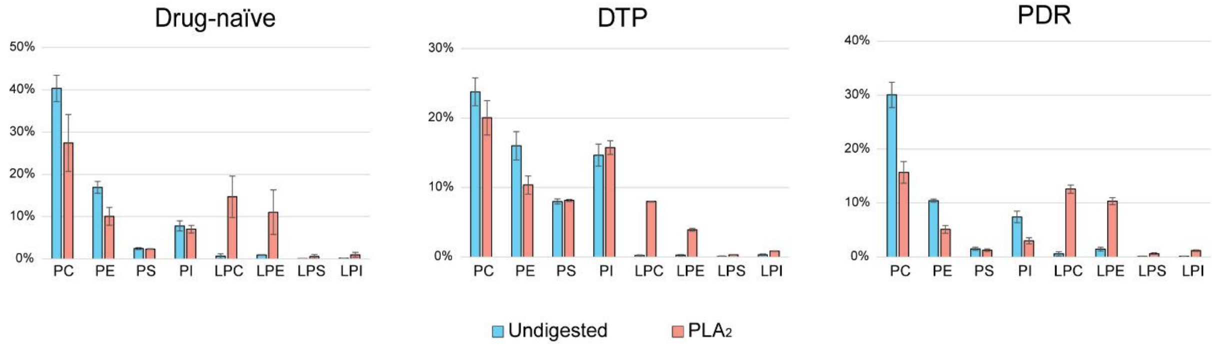

Supplementary Figure 1. Comparison of undigested phospholipids and lipids digested with phospholipase A_2. Bar graphs show relative proportions of common phospholipids (PC, PE, PS, PI) and lysophospholipids (LPC, LPE, LPS, LPI) found in WM164 cells in drug-naïve, drug-tolerant persister and permanent drug-resistant states. Data represent lipid species quantified from untreated cells and cells following digestion of the outer leaflet with phospholipase A_2 (PLA_2). Digested samples showed an increased relative proportion of lysophospholipids compared with undigested samples. Mean ± SD of three technical replicates of one biological replicate are shown.

A

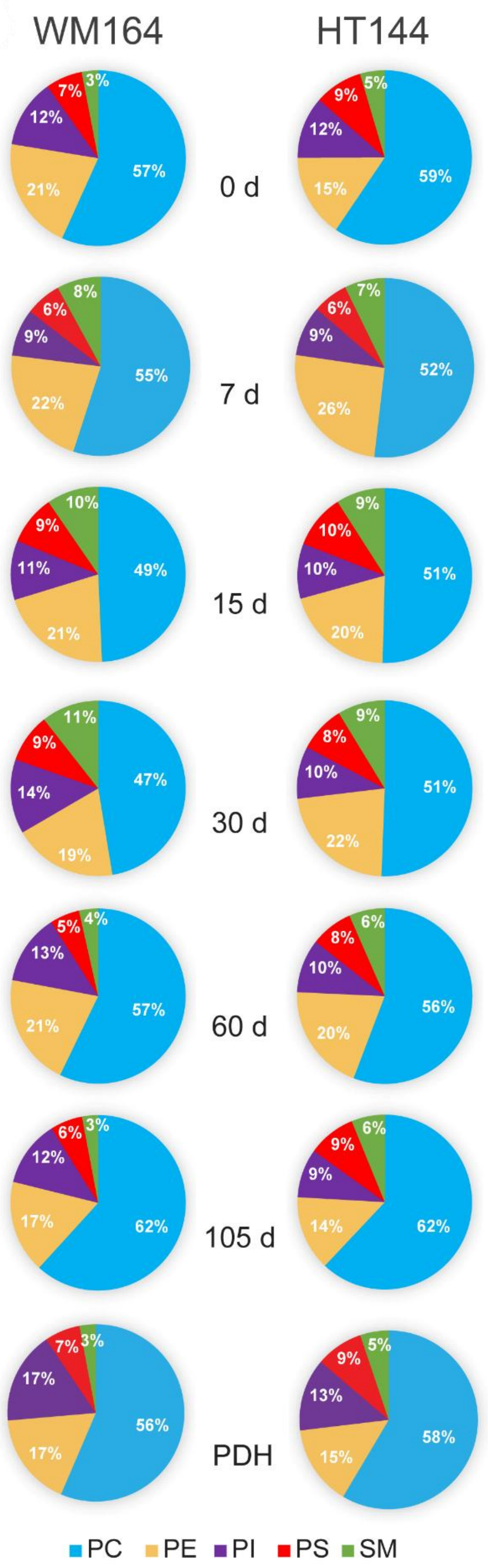

B

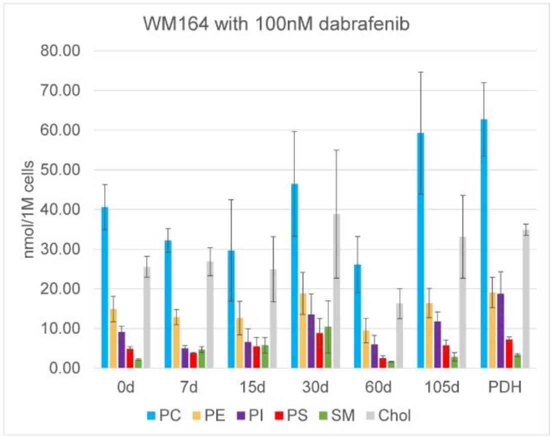

WM164 with dabrafenib (100 nM)

		0d	7d	15d	30d	60d	105d	PDH
nmol/1M cells	PC	40.60	32.20	29.68	46.45	26.13	59.28	62.74
	PE	14.89	12.85	12.62	18.83	9.49	16.40	19.08
	PI	9.04	5.00	6.60	13.47	5.96	11.73	18.75
	PS	4.84	3.82	5.51	8.85	2.51	5.70	7.25
	SM	2.17	4.67	5.78	10.40	1.61	2.85	3.30
	Chol	25.56	26.85	24.93	38.84	16.28	33.12	34.89
SD	PC	5.69	2.98	12.81	13.23	7.06	15.39	9.21
	PE	3.22	1.91	4.22	5.30	3.11	3.71	3.83
	PI	1.49	0.67	3.30	5.23	2.30	2.44	5.56
	PS	0.59	0.22	2.23	3.65	0.61	1.42	0.62
	SM	0.18	0.73	1.98	6.56	0.12	1.06	0.33
	Chol	2.64	3.54	8.20	16.12	3.81	10.44	1.43

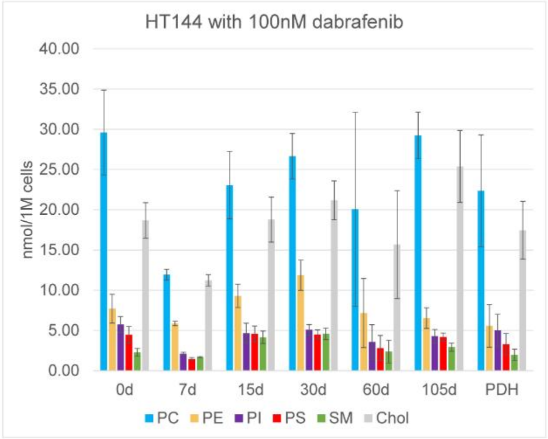

HT144 with dabrafenib (100 nM)

		0d	7d	15d	30d	60d	105d	PDH
nmol/1M cells	PC	29.61	11.94	23.04	26.65	20.05	29.25	22.35
	PE	7.70	5.86	9.30	11.86	7.16	6.54	5.56
	PI	5.74	2.09	4.66	5.07	3.55	4.28	5.02
	PS	4.46	1.47	4.57	4.50	2.81	4.17	3.28
	SM	2.27	1.67	4.15	4.59	2.37	2.91	1.97
	Chol	18.69	11.23	18.78	21.17	15.67	25.38	17.46
SD	PC	5.26	0.66	4.18	2.83	12.04	2.90	6.95
	PE	1.79	0.28	1.45	1.87	4.29	1.29	2.65
	PI	0.98	0.21	1.22	0.65	2.16	0.84	1.99
	PS	1.05	0.17	0.98	0.56	1.57	0.49	1.34
	SM	0.49	0.08	0.77	0.71	1.41	0.51	0.70
	Chol	2.20	0.70	2.80	2.42	6.70	4.47	3.59

Supplementary Figure 2. Lipid composition of WM164 and HT144 cells while acquiring resistance to dabrafenib. Related to Figure 2. (A) Pie charts show average proportion (mol%) of the four major glycerophospholipid classes in mammalian cell membrane (i.e., PC-, PE-, PI-, and PS-) and of sphingomyelin (SM) in WM164 and HT144 cells during treatment with dabrafenib for 0, 7, 15, 30, 60, 105 days and 15 days without drug or on "post-drug holiday" (PDH). (B) Absolute quantification of each lipid class in nmol per 1 million cells. Data represented as mean ± SD (n = 3 biological replicates).

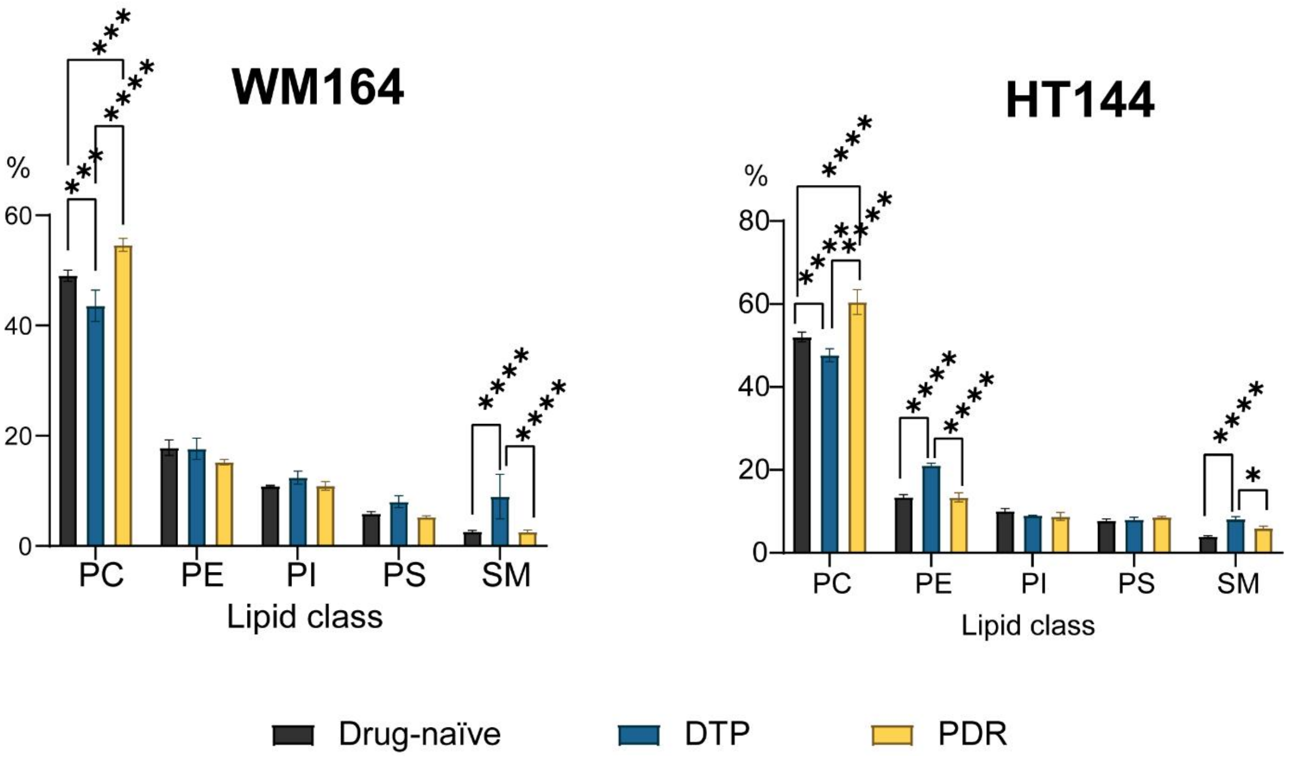

Supplementary Figure 3. Proportion of phospholipids detected in drug-naïve, drug-tolerant and permanent drug-resistant melanoma cells. Related to Figure 2A. Histograms show proportions of the four major glycerophospholipid classes (i.e., PC-, PE-, PI-, and PS-) and of sphingomyelin (SM) in WM164 and HT144 cells during their drug-naïve, drug-tolerant (DTP) and permanent drug-resistant (PDR) states. 2-way ANOVA was performed for statistical analyses with Tukey's multiple comparison test. *P*-values ≤0.05 (*), ≤0.01(**), ≤0.001 (***), ≤0.0001(****) are indicated to show statistical significance level between two states linked by a bracket. All other comparisons were not significant.

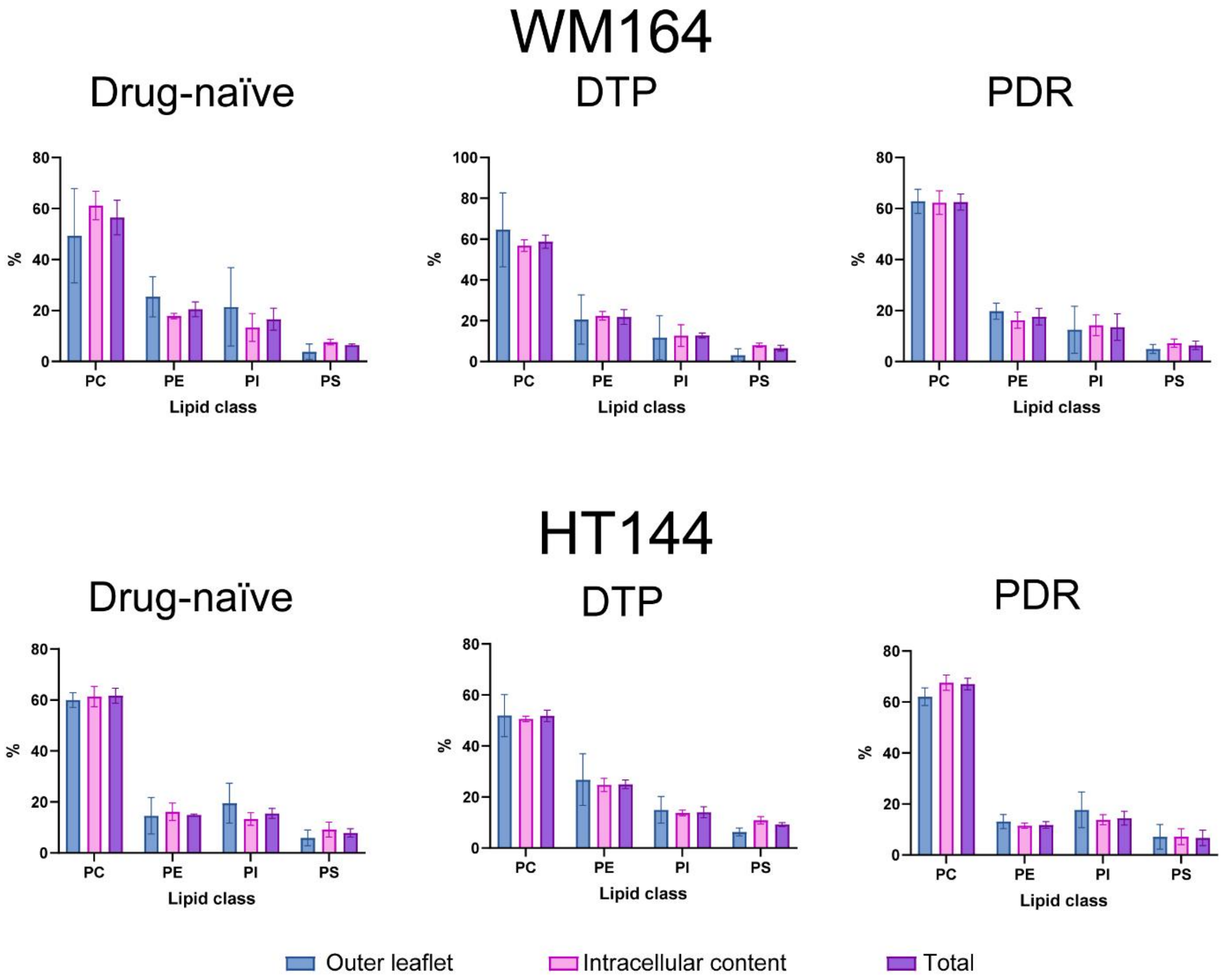

Supplementary Figure 4. Comparison of the proportion of phospholipids in whole-cell lipid extracts, outer leaflet and intracellular content within each cell state. Related to Figure 4A. Histograms show proportions of the four major glycerophospholipid classes (i.e., PC-, PE-, PI-, and PS-) obtained in whole cell extracts (total), in the outer leaflet of the cell membrane (outer leaflet), and in the inner cytoplasmic leaflet + intracellular organelles (intracellular content) for drug-naïve, drug-tolerant (DTP) and permanent drug-resistant (PDR) states of WM164 and HT144 cells. 2-way ANOVA was performed for statistical analyses with Tukey's multiple. Differences within each lipid class for individual cell states are not significant.

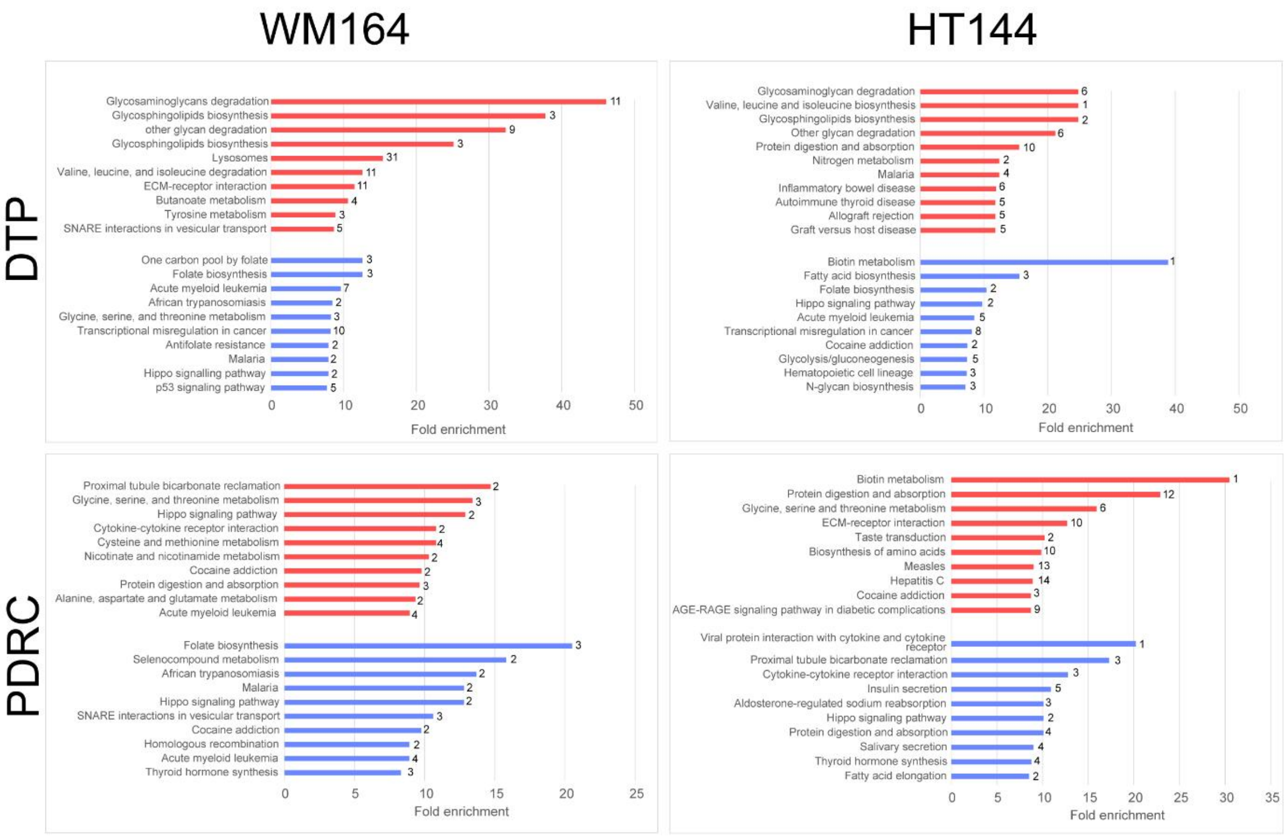

Supplementary Figure 5. KEGG pathway WM164 and HT144 cells treated with 100nM dabrafenib. Diagrams represent the number (end of each bar) of upregulated (red) and downregulated (blue) proteins involved in identified KEGG pathways (https://www.genome.jp/kegg/pathway.html) with P-value ≤ 0.05; log2 fold change ± 1. (fold enrichment: enrichment factor calculated as a quotient of number of found protein and number of expected proteins).

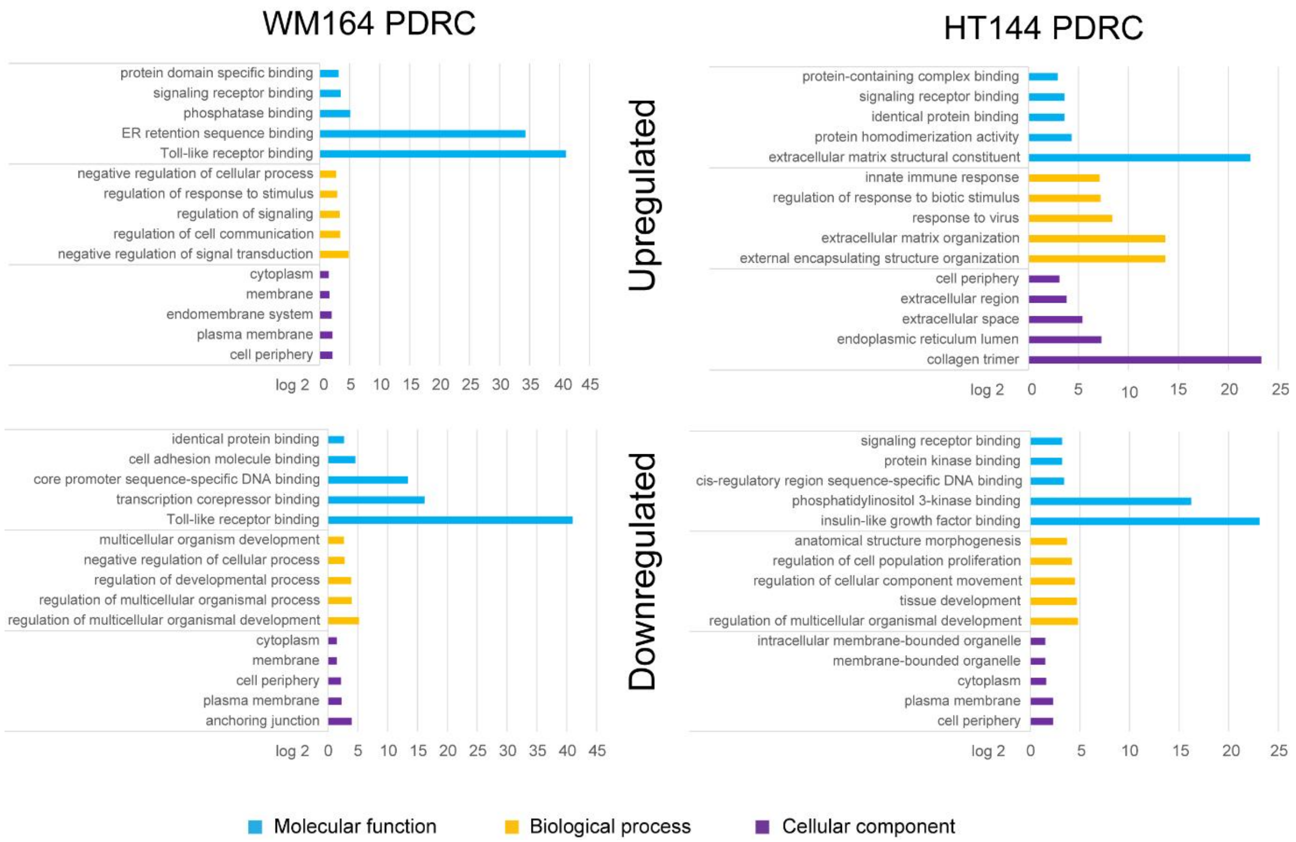

Supplementary Figure 6. Gene ontology of permanent drug-resistant WM164 and HT144 cells. Gene ontology functional analysis of differentially expressed proteins in WM164 and HT144 permanent drug-resistant cells (PDRC). Proteins from PDRC WM164 and HT144 cells were compared to proteins from drug-naïve cells. Histograms represent fold enrichment of top 5 differentially expressed proteins (P-value ≤ 0.05; log2 fold change ± 1) involved in cellular component (purple), biological process (yellow) and molecular function (blue) categories.
